# Long-Term Warming Shifts the Composition of Bacterial Communities in the Phyllosphere of *Galium album* in a Permanent Grassland Field-Experiment

**DOI:** 10.3389/fmicb.2018.00144

**Published:** 2018-02-13

**Authors:** Ebru L. Aydogan, Gerald Moser, Christoph Müller, Peter Kämpfer, Stefanie P. Glaeser

**Affiliations:** ^1^Institute for Applied Microbiology, Justus Liebig University Giessen, Giessen, Germany; ^2^Institute for Plant Ecology, Justus Liebig University Giessen, Giessen, Germany; ^3^School of Biology and Environmental Science, University College Dublin, Dublin, Ireland

**Keywords:** global climate change, warming experiment, phyllosphere, *Sphingomonas*, *Enterobacteriaceae*, *Buchnera*, grassland

## Abstract

Global warming is currently a much discussed topic with as yet largely unexplored consequences for agro-ecosystems. Little is known about the warming effect on the bacterial microbiota inhabiting the plant surface (phyllosphere), which can have a strong impact on plant growth and health, as well as on plant diseases and colonization by human pathogens. The aim of this study was to investigate the effect of moderate surface warming on the diversity and composition of the bacterial leaf microbiota of the herbaceous plant *Galium album*. Leaves were collected from four control and four surface warmed (+2°C) plots located at the field site of the Environmental Monitoring and Climate Impact Research Station Linden in Germany over a 6-year period. Warming had no effect on the concentration of total number of cells attached to the leaf surface as counted by Sybr Green I staining after detachment, but changes in the diversity and phylogenetic composition of the bacterial leaf microbiota analyzed by bacterial 16S rRNA gene Illumina amplicon sequencing were observed. The bacterial phyllosphere microbiota were dominated by *Proteobacteria*, *Bacteroidetes*, and *Actinobacteria*. Warming caused a significant higher relative abundance of members of the *Gammaproteobacteria*, *Actinobacteria*, and *Firmicutes*, and a lower relative abundance of members of the *Alphaproteobacteria* and *Bacteroidetes*. Plant beneficial bacteria like *Sphingomonas* spp. and *Rhizobium* spp. occurred in significantly lower relative abundance in leaf samples of warmed plots. In contrast, several members of the *Enterobacteriaceae*, especially *Enterobacter* and *Erwinia*, and other potential plant or human pathogenic genera such as *Acinetobacter* and insect-associated *Buchnera* and *Wolbachia* spp. occurred in higher relative abundances in the phyllosphere samples from warmed plots. This study showed for the first time the long-term impact of moderate (+2°C) surface warming on the phyllosphere microbiota on plants. A reduction of beneficial bacteria and an enhancement of potential pathogenic bacteria in the phyllosphere of plants may indicate that this aspect of the ecosystem which has been largely neglected up till now, can be a potential risk for pathogen transmission in agro-ecosystems in the near future.

## Introduction

Global warming is an ongoing climate change effect and will lead to an increase of the mean global air temperature by 2–3°C until 2050 as predicted by the Intergovernmental Panel on Climate Change (IPCC, 2013). Elevated surface temperature can affect plants and their symbiotic microbiota (the plant holobiont; Margulis, 1993; Vandenkoornhuyse et al., 2015) by influencing plant growth, health, and yield (Wu et al., 2011; Jansen-Willems et al., 2016). An important plant-associated habitat colonized by specific bacterial communities is the phyllosphere, the aerial part of plants dominated by leaves (Vorholt, 2012; Bringel and Couée, 2015). Estimated densities of leaf surface colonizing bacteria (epiphytic bacteria) range between 10^6^ and 10^7^ per cm^2^ leaf surface (Vorholt, 2012). The composition of the phyllosphere microbiota can vary among plant species and is influenced by the geographical location, season, and different environmental conditions including temperature, rain, sunlight exposure, dryness (Whipps et al., 2008; Knief et al., 2010; Wellner et al., 2011; Rastogi et al., 2012; Copeland et al., 2015; Ding and Melchner, 2016). Phyllosphere inhabiting bacteria can be beneficial, pathogenic, or antagonistic (plant-protecting) for the host plant and can strongly contribute to plant health and yield by complex plant–microbe interactions (Vorholt, 2012; Bulgarelli et al., 2013; Brader et al., 2017). So far, only a few studies have investigated the effects of increasing temperature on the phyllosphere microbiota, beneficial microbes (Compant et al., 2010), richness of bacteria and fungi in the phyllosphere (Peñuelas et al., 2012), composition of the phyllosphere endophytic microbiota and the plant metabolism (Campisano et al., 2014). Furthermore, the combination of both elevated temperature and CO_2_ led to an increase or decrease of specific phyllosphere inhabiting bacteria (Ren et al., 2015). Campisano et al. (2017) showed a shift of the endophytic community of *Vitis vinifera* induced by temperature changes and a stronger influence of the season, on bacterial taxa in stems (phyllosphere compartment) compared to roots. Respective seasonal shifts of the leaf microbiota were also shown in several other studies, e.g., by Redford and Fierer (2009), Ding and Melchner (2016). This demonstrates the higher sensitivity of the phyllosphere to temperature changes and illustrates the effects of climate changes. Agler et al. (2016) introduced the microbial “hub” – concept into the research on microbe–microbe interactions in the phyllosphere (van der Heijden and Hartmann, 2016). Based on this concept, specific highly interactive bacterial taxa play a key role in the phyllosphere microbiota by their interactions with several other community members. Changes in the relative abundance of those “hub” microbes induced by abiotic or host factors, like climate, distribution, or host resistance alleles, can strongly affect the total microbial community structure. Warming is one of the environmental factors, that can affect hub microbes and thus strongly impact the phyllosphere microbiota. This has not been studied in depth so far.

An increasing number of studies showed that the phyllosphere of plants can be efficiently colonized by human pathogens (Mootian et al., 2009; Oliveira et al., 2011, 2012; Holden et al., 2015). This is also critical due to the fact, that a high number of potentially pathogenic bacteria, including the possibility that some of them may be resistant to antibiotics, can be released into the environment from livestock husbandry and wastewater treatment plants (Heuer et al., 2011; Schauss et al., 2015, 2016).

Previous studies have shown, that moderate warming (+2°C) was more beneficial for plants whereas higher temperatures had adverse effects (Jin et al., 2011; Martinez et al., 2014). The effect of warming on different plant–insect interactions was investigated in several studies (Dong et al., 2013; Berthe et al., 2015; Kozlov et al., 2017). Studies on the effects of warming on plant–bacteria interactions were mainly focused on soil microbial communities (Sheik et al., 2011; DeAngelis et al., 2015; Romero-Olivares et al., 2017), thus little is known about the phyllosphere associated microbial communities. The impact of elevated temperature on the phyllosphere microbiota in long-term experiments to our knowledge is so far only studied by Ren et al. (2015) in the rice phyllosphere using a 16S rRNA gene amplicon pyrotaq sequencing approach.

Worldwide, grasslands cover approximately 26% of the world land area (Freibauer et al., 2004; Conant, 2010) and constitute an important ecosystem function including food for livestock and wild animals. However, long-term warming effects on the phyllosphere microbiota of a permanent grassland have not been studied so far.

In a 6.5-year experiment examining the effects of increasing the leaf surface temperature (by +2°C), an increase in the aboveground biomass was observed (Jansen-Willems et al., 2016). This experiment was used to get a first insight on the effect of a moderate, (+2°C) long-term warming on the composition of the phyllosphere inhabiting microbial community of the most abundant forb, *Galium album* Mill., within the permanent grassland. Total cell counts and bacterial 16S rRNA gene amplicon sequencing analyses using the Illumina sequencing technology were used to monitor these effects. This study was performed to test the hypothesis that long-term moderate (+2°C) surface warming affects the phyllosphere microbiota with respect to (1) the total abundance of phyllosphere bacteria and (2) the diversity and phylogenetic composition of the bacterial communities in the phyllosphere.

## Materials and Methods

### Field Site Description

The experiment was established on 24th January 2008 on the permanent temperate grassland of the Environmental Monitoring and Climate Impact Research Station at Linden in Germany and was run until 12th May 2014. The field site is located at 50°31.6′N and 8°41.7′E and sits 172 m above sea level. The mean annual temperature is 9.4°C and the mean cumulative annual precipitation is 558 mm (1998–2013). The mean winter (December–February) temperature over the period 2008–2014 was 1.4°C ± 5.1 and the mean summer (June–August) temperature over the corresponding time period was 17.6°C ± 5.2. The vegetation is characterized as a seminatural grassland dominated by *Arrhenatheretum elatioris* and *Filipendula ulmaria*. Among the forbs present in the grassland, *G. album* was the dominating species. The permanent grassland was treated like a meadow and was routinely cut twice a year from 1993 onward. *G. album* did then not grow over the autumn or winter months. Since 1995, 40 kg N ha^-1^ per year was applied each spring. No plowing has been performed at the experimental site for more than 100 years. The soil on the field site was classified as a Fluvic Gleysol on loamy-sandy sediments over a clay layer.

### Experimental Setup

A 100 m^2^ site area was divided into 16 equally sized plots, arranged in four rows of four plots for the experiment (Supplementary Figure 1). The plots were set up according to a Latin square (each treatment occurred once in each row and each column) in four randomly distributed plot replicates. The control and treated plots both appeared twice in each replicate. Over the “treated plots” an IR-lamp was set up to elevate the surface temperature of the plants and the soil resulting in a mean increase of surface temperature of +2°C (mean 1.9 standard error 0.03, measured 5 cm above soil). The temperature treatment was performed with Edison screw base ceramic infrared heaters of 230V and 250W with reflector and E27 ceramic lamp holder (Friedrich Freek GmbH, Menden, Germany). To avoid the displacement of the lamps (by external force) they were stabilized with three metal bars, and a metal plate above the lamps was used as rain protection. A metal plate for rain protection was also installed above the control plots for comparability to the elevated temperature treatments. The experiment ran for 6.5 years (2008–2014).

The area in the center of each plot (318 cm^2^) directly underneath the IR-lamp was used for sampling. In order to prevent interference between the control and treated plots, the areas of sampling were 2.5 m apart from each other.

### Sample Collection

Leaf samples of the herbaceous plant *G. album* (dicot) were taken in the morning of 12th May 2014. All four replicates of the elevated temperature and control plots were sampled separately. Using a sterile pestle, wreaths of leaves were collected in a 540-mL sterile whirl-pack bag (Carl Roth GmbH, Karlsruhe, Germany) and directly stored in a dark precooled (-80°C) transport box. Samples for further molecular analyses were stored at -80°C immediately after they arrived at the laboratory.

### Total Cell Counts

The concentration of leaf-attached bacterial cells per gram of leaf fresh weight (FW) was determined by SybrGreen I (SG-I) staining using the method of Lunau et al. (2005). For this 4–6 g of leaf material of *G. album*, was placed in 50-mL falcon tubes and 30 mL autoclaved phosphate buffered saline (PBS; 130 mM NaCl, 7 mM Na_2_HPO_4_, 3 mM NaH_3_PO_4_ per liter; pH 7) was added to the samples. The tubes were shaken for 10 min to detach bacterial cells using a vertical shaker (level 8/Edmund Bühler, Tübingen). The detachment was repeated by a second washing step using an additional 30 mL PBS buffer. Both buffer fractions were combined for further analysis. Detached cells were fixed with 2% (v/v) glutaraldehyde (AppliChem) and the cells were stained with SYBR Green-I (SG-I; Sigma-Aldrich) solution. Fixed cells (filtered volume: 0.7 mL) were therefore collected on black polycarbonate filters (0.2 μm pore size, Millipore, Eschborn, Germany) using a SG-I moviol staining solution (Lunau et al., 2005). By epifluorescence microscopy at 1000× magnification with a Leica DFC 3000G microscope (Leica, Germany) filters were analyzed using the LAS X software for cell measurement. SG-I stained cells were counted from 10 digital images, which were generated with a Leica DFC 3000G (Leica, Germany) camera system. Mean values of the four plots were calculated from the mean values of the 10 picture counts. The respective standard deviation was considered by error propagation. The presence of statistically significant differences between treatments were measured with the Student’s *t*-test in SigmaPLOT 11 (Systat Software Inc.).

### DNA-Extraction

Total DNA-extraction from whole frozen leaves was performed with the genomic DNA extraction kit for soil, the NucleoSpin^®^ Soil Kit (Macherey Nagel, Germany). Approximately 170 mg of whole leaves were weighed and extracted according to manufactures instructions. Total leaves were used for DNA extraction in order to ensure that bacterial cells strongly attached to the leaf surface and partially embedded in a biofilm (“phylloplane”) were include in the analysis. This was done in order to avoid the exclusion of the most active fraction of the phyllosphere microbiota. Endophytic bacteria were only partially co-extracted, because leaves remained intact after the first steps of DNA extraction. Extraction was started using lysis buffer SL1 and afterward steps 1–5 were repeated with lysis buffer SL2, thus the leaves were lysed twice. The supernatants were loaded on one NucleoSpin Soil Column for DNA binding. The silica membrane was centrifuged twice for washing and the DNA was eluted in two subsequent elution steps with 25 μL PCR water. The DNA containing eluates were combined and DNA quality and quantity were investigated using a NanoDrop spectrophotometer (Thermo Scientific).

### 16S rRNA Gene Amplicon Illumina MiSeq Analysis

The diversity and phylogenetic composition of the bacterial communities were analyzed by 16S rRNA gene amplicon sequencing using universal bacterial 16S rRNA gene V5-V6 region targeting primers 799F (5′-AACMGGATTAGATACCCKG-3′) and 1115R (5′-AGGGTTGCGCTCGTTG-3′) which are recommended for the exclusion of chloroplast DNA amplification (Chelius and Triplett, 2001; Redford et al., 2010). The PCR amplification and Illumina 300 bp paired-end read sequencing was done by LGC Genomics (Berlin, Germany) with the Illumina MiSeq V3 system. Sequence libraries were demultiplexed with the Illumina bcl2fastq 1.8.4 software, reads were sorted by amplicon inline barcodes. Reads with missing barcodes, one-sided barcodes or conflicting barcode pairs were discarded. Barcodes, adaptors and primer sequences were clipped after sorting. Reads with a final length <100 bp after adaptor clipping were discarded. Sequences were oriented into the forward and reverse primer direction. Forward and reverse reads were combined using BBMerge 34.48^1^. Datasets of combined reads were analyzed in the SILVAngs analysis pipeline^2^. Using the SILVA Incremental Aligner [SINA version 1.2.10 for ARB SVN (revision 21008)] (Pruesse et al., 2012) all sequence reads were aligned against the SILVA SSU rRNA SEED database and quality controlled (Quast et al., 2013). Reads with more than 2% of ambiguities or 2% of homopolymers, respectively, were excluded from the analysis. Putative contaminations and artifacts, reads containing a low alignment quality (50 alignment identity, 40 alignment score reported by SINA) were detected and excluded from downstream analysis. After this initial quality control step, dereplication and clustering was performed with cd-hit-est (version 3.1.2^3^) (Li and Godzik, 2006) using *accurate mode*, disregarding overhangs, and using identity criteria of 1.00 and 0.98, respectively. The classification was done with the nucleotide BLAST search against the non-redundant version of the SILVA SSU Ref dataset (release 123^4^) using standard settings (Camacho et al., 2009) of blastn (version 2.2.30+^5^). Each operational taxonomic unit (OTU) reference read classification was mapped onto all reads that were assigned to the respective OTU. This resulted in quantitative information (number of individual reads per taxonomic path), within the limitations of PCR and sequencing technique biases, as well as, multiple rRNA operons. Reads missing any BLAST hits or reads with weak BLAST hits remained unclassified, where the function “(% sequence identity + % alignment coverage)/2” did not pass the value of 93. Then assignment of these reads to a meta group of “No Relative” in the SILVAngs fingerprint and Krona charts (Ondov et al., 2011) were done. This method was first applied in the studies published by Ionescu et al. (2012) and Klindworth et al. (2013). Archaeal, chloroplast, mitochondria, and “No Relative” reads were excluded from the analysis. Only reads assigned to the bacterial phyla were used for further analysis. Amplicon sequence data were submitted to the sequence read archive (SRA) of the NCBI within the BioProject PRJNA400850 as BioSamples SAMN07562291 to SAMN07562298.

### Statistical Analysis

Statistical analyses were performed by PAST software version 3.11 (Hammer et al., 2001). Non-metric multidimensional scaling (nMDS; Taguchi and Oono, 2005) and principal component analysis (PCA; Davis, 1986; Harper, 1999) were used to display the differences between the relative abundance pattern of leaf associated bacteria and the influence of the individual phyla or taxa to the differences between treatments. The differences between the control and treated plots were tested for statistical significance using a one-way ANOSIM (Clarke, 1993). nMDS and one-way ANOSIM analysis based on similarity matrices was calculated with the Bray–Curtis similarity index. SIMPER analyses (Clarke, 1993) were used to detect phyla and taxa of lower ranks, which contributed most to the differences between treatments using the same similarity index. Richness and diversity of the phyllosphere communities were calculated with Chao 1, Shannon, evenness and dominance indices (Harper, 1999) and rarefaction curve analyses (Krebs, 1989) considering the number of taxonomic paths and the number of individual reads per taxonomic path. Boxplots of cell counts, of the taxa contributing to the main differences and of the diversity indices were performed in SigmaPLOT 12.5 (Systat Software Inc.) and the significance of the differences between C and T plots were tested using Student’s *t*-test with the same software.

## Results

### Bacterial Abundance in the Phyllosphere of *G. album* of Control and Warmed Plots

**FIGURE 1:**
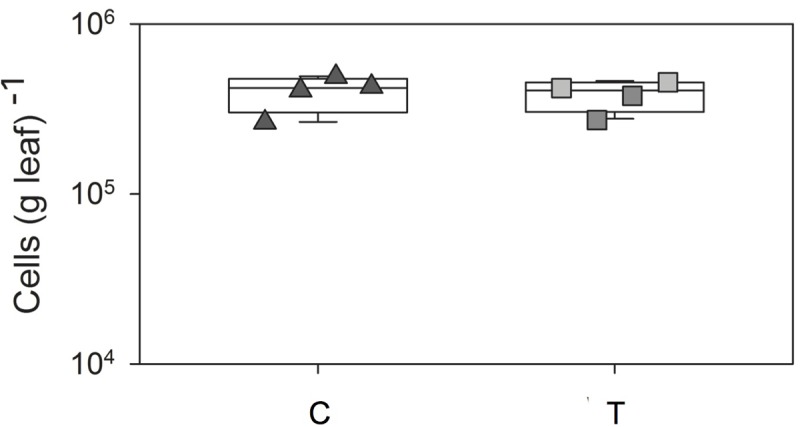
Box-plots of total cell number counts per g *G. album* leaf of C and T plots. C = ambient temperature (C), T = elevated temperature (T). Squares = four C plots, triangles = four T plots, box-plots were performed in SigmaPLOT (Applied Maths).

The numbers of leaf-associated bacteria (stained by Sybr Green I after detachment from leaves) observed in leaves sampled randomly from *G. album* control plants (ambient temperature) (control plots; C 1–4) and the those on leaves exposed to elevated temperature (+2°C; elevated temperature plots; T 1–4) is shown in Supplementary Figure 1. The bacterial cell concentration was in the range of 10^5^ cells g^-1^ leaf fresh weight (FW) for *G. album* leaves of C and T plots (**Figure 1** and Supplementary Table 1). Elevated temperature had no significant effect on the abundance of leaf-associated bacteria (*p* > 0.05, Student’s *t*-test). Cell morphologies of bacterial cells detached from leaves collected from C and T plots were also similar. Most of the detached cells were rod-shaped with a mean size of 1.6 (±0.4) × 0.6 (±0.2) μm (C) and 1.7 (±1.1) × 0.6 (±0.2) μm (T), respectively.

### Analysis of the Phylogenetic Composition of the Bacterial Phyllosphere Microbiota

Bacterial 16S rRNA gene amplicon sequencing was performed from total DNA extracts obtained from *G. album* leaves, which were randomly collected from the four C and T plots. A total number of 638,626 paired end sequences with an average sequence length of 299 nucleotides (nt) were obtained from the eight analyzed leaf samples derived from eight plots. In summary, 204,704 sequences (32.1%) were rejected, because they failed the quality control of the SilvaNGS pipeline. Finally, 433,922 sequences (7,627–88,073 per sample) were subjected to analysis (Supplementary Table 2). A total number of 14,420 OTUs (2.3%) was obtained based on a 98% sequence similarity threshold from 59,031 clustered sequences (9.3%) including 360,471 replicates (56.4%). Respective OTUs were assigned to taxonomic paths (named as “phylogenetic groups”) with a maximum resolution at the genus level. The relative abundance of chloroplast and mitochondrial 16S rRNA gene sequence was in the range of 1.9–34.4% and 0.5–12.3% for individual samples, respectively (Supplementary Figure 2). This was in the range reported also in other phyllosphere studies (Lopez-Velasco et al., 2011; Ferrando et al., 2012; Ren et al., 2015). All sequences which were identified as *Archaea* (304 sequences, 0.1%), chloroplasts (113,103 sequences; 19.2%), mitochondria (42,530 sequence, 7.2%) or which did not match any known taxa (sequence similarity < 93% to the next known taxa; summarized as “no relative”; 0.1%) were excluded from the analysis (Supplementary Figure 2). Only sequences assigned to the domain *Bacteria* were considered subsequently. Respective sequences were set to 100%.

### Elevated Temperature Effects on the Composition and Diversity of the Phyllosphere Microbiota

**FIGURE 2:**
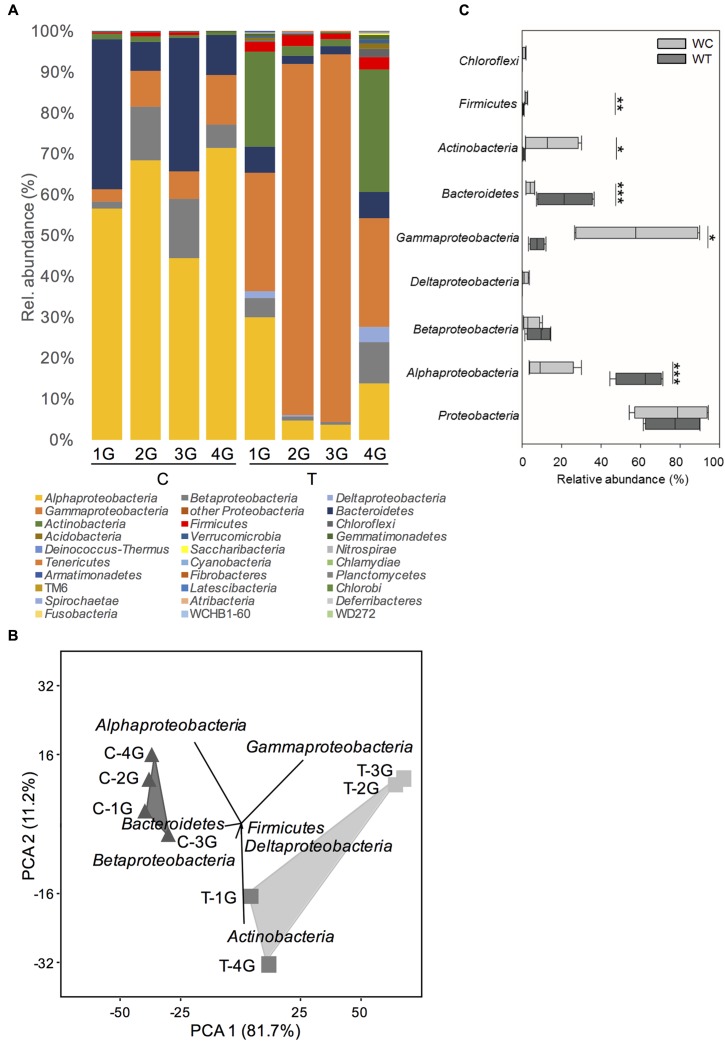
Phylogenetic composition of phyllosphere inhabiting bacteria of *Galium album* leaves from plots with ambient temperature (C 1–4) and +2°C surface temperature elevated plots (T 1–4) resolved at the level of *Bacteria* phyla. **(A)** Relative abundance of different bacterial phyla on *G. album* leaves from C and T plots. **(B)** Principal component analysis (PCA) of relative abundance pattern of respective bacterial communities based on the phyla assignment. Eigenvalues for the compared principal components are given in brackets (%) at the respective axes of the graphs. The contribution of different taxonomic groups to the placement of the samples in the PCA plots are indicated as biplots in the graph, and **(C)** variation of the relative abundances of the most abundant phyla among *G. album* leaf samples from C and T plots. Asterisks are representing statistical significance: ^∗^*p* < 0.05; ^∗∗^*p* < 0.01; ^∗∗∗^*p* < 0.001. G = *Galium album*; C = ambient temperature; T = elevated temperature; 1–4 = biological replicates.

In summary, 28 different phyla or Candidatus phyla (18 from C and 26 from T plots) were detected in the phyllosphere of *G. album* leaves. Bacterial communities of both treatments were dominated by *Proteobacteria* including mainly *Alpha*-, *Gamma*-, *Beta*-, and *Deltaproteobacteria*, followed by *Bacteroidetes* and *Actinobacteria* (**Figure 2A**). Lower abundant phyla (contribution > 1%) detected in the phyllosphere of *G. album* leaves from C as well T plots, were *Firmicutes*, *Chloroflexi*, and *Acidobacteria* (**Figure 2A** and Supplementary Table 3).

Principal component analysis (**Figure 2B**) of the relative abundance patterns of bacterial phyla showed a distinct separation of the bacterial phyllosphere microbiota on *G. album* leaves of C and T plots (*p* = 0.03; one-way ANOSIM). According to those samples PCA biplots indicated, that changes in the relative abundance of *Alphaproteobacteria*, *Gammaproteobacteria*, *Bacteroidetes*, and *Actinobacteria* had the main impact on the differences between the bacterial phyllosphere microbiota of *G. album* leaves from C and T plots. *Alphaproteobacteria*, *Betaproteobacteria*, and *Bacteroidetes* were more abundant in control plots, while *Gammaproteobacteria, Deltaproteobacteria*, *Actinobacteria*, and *Firmicutes* were more abundant in warmed plots (**Figure 2B**).

Considering the highly diverse phylum *Proteobacteria* as one group, no significant differences were obtained with respect to relative abundance between control and warmed plots (**Figure 2C**). However, if most abundant classes within the *Proteobacteria* (representing >99% of all *Proteobacteria* sequences per sample) were considered separately (**Figure 2C**) significant differences were obtained for the relative abundance of *Alphaproteobacteria* and *Gammaproteobacteria*, but no significant differences were seen for *Betaproteobacteria* and *Deltaproteobacteria* between C and T plots. The relative abundance of *Alphaproteobacteria* was significantly lower under warmed conditions (C: 60.1 ± 10.6%; T: 13.0 ± 10.6%), while the relative abundance of *Gammaproteobacteria* significantly increased under warmed conditions (C: 7.6 ± 3.2%; T: 57.9 ± 30.2%). The relative abundance of *Betaproteobacteria* was slightly but not significantly lower, while the relative abundance of *Deltaproteobacteria* was slightly but not significantly higher in warmed plots.

A significantly lower relative abundance of *Bacteroidetes* (C: 21.6 ± 13.1%; T: 4.1 ± 2.1%) and a significantly higher relative abundance of *Actinobacteria* (C: 1.0 ± 0.5%; T: 14.3 ± 12.6%) were detected on leaves from T compared to C plots (**Figure 2C**). A phylum with lower abundance that was affected by warming was *Firmicutes* (C: 0.6 ± 0.4%; T: 2.3 ± 0.6%), which was detected in significant higher relative abundance on leaves derived from T plots (**Figure 2C**).

For a more detailed insight into the effects of warming, the diversity and composition of the bacterial phyllosphere microbiota was analyzed at a higher phylogenetic resolution by comparing the relative abundance patterns of phylogenetic groups (mainly defined at the genus level). In total, 724 different phylogenetic groups (177–502 per sample) were detected (Supplementary Table 4). On average 9 (±1) and 12 (±6) phylogenetic groups had a relative abundance higher than 1% in leaf samples of C and T plots, respectively.

**FIGURE 3:**
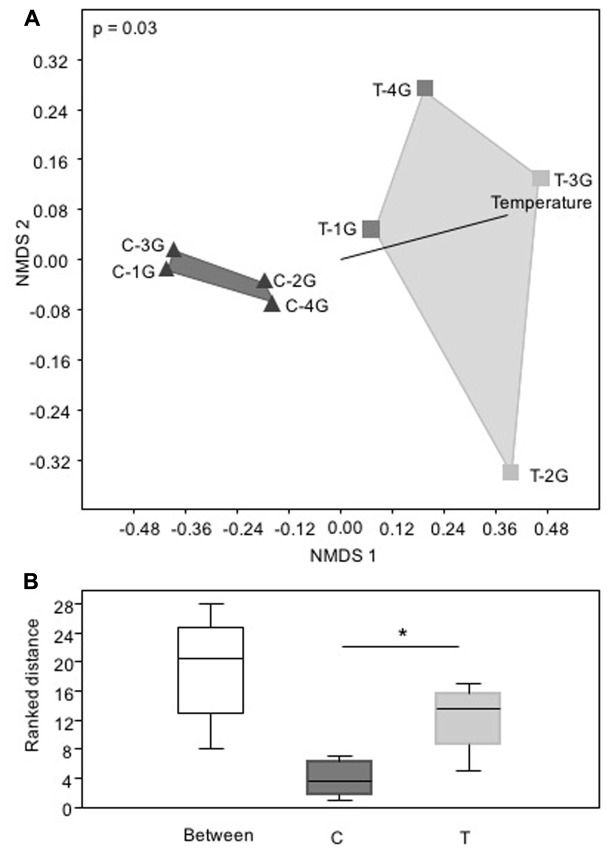
Phylogenetic composition of phyllosphere inhabiting bacteria of *Galium album* leaves from plots with ambient temperature (C 1–4) and +2°C surface temperature elevated plots (T 1–4) with a resolution at the level of *Bacteria* taxa. **(A)** Non-metric multidimensional scaling (nMDS) analysis of relative abundance pattern of respective bacterial communities based on the taxa assignment, calculated with Bray–Curtis similarity matrix, and **(B)** Rank differences of the leaf microbiota from C and T plots analyzed at the level of bacterial taxa. Analysis was performed in PAST3 and based on a Bray–Curtis similarity matrix calculation. Box-plots were calculated with the interpolated quartile method. Outliers were not determined. Significant differences were determined by one-way ANOSIM. Asterisk is representing statistical significance: ^∗^*p* < 0.05.

Non-metric multidimensional scaling of bacterial community patterns based on Bray–Curtis similarities revealed significant differences (corrected *p* ≤ 0.03, one-way ANOSIM) between the bacterial community compositions of C compared to T leaves (**Figure 3A**). The inclusion of temperature as an environmental variable to the nMDS analysis revealed, that surface temperature correlated with the significant differences of the community patterns of the leaf microbiota derived from C and T plots (**Figure 3A**). The nMDS plot and the corresponding ranked distance analysis of the bacterial community patterns (**Figures 3A,B**) showed a higher variability (relative abundance patterns of different phylogenetic groups) obtained from leaf samples of the four T plots, but were very similar to the bacterial communities of the C plots. This indicated that leaf associated bacterial communities were much more stable under control than elevated temperature conditions.

**FIGURE 4:**
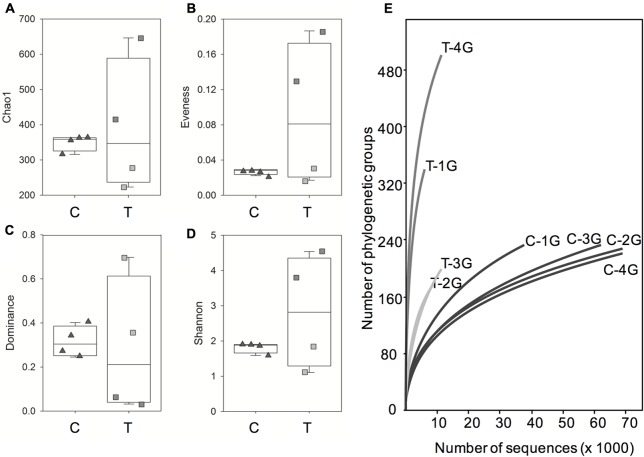
Box-plots of alpha diversity indices Chao 1 **(A)**, evenness **(B)**, dominance **(C)** and Shannon index **(D)** and rarefaction curves **(E)** for the phyllosphere community of *Galium album* leaves from C and T plots. C = ambient temperature (C), T = elevated temperature (T). Triangles = four C plots, squares = four T plots. **(E)** Rarefaction curves (specimens versus taxa) based on Illumina 16S rRNA gene amplicon sequencing of microbial communities from all C (C 1–4) and T (T 1–4) plots.

This finding was supported by the comparison of bacterial communities using different diversity measurements (**Figures 4A–E**). Diversity indices were similar for the bacterial phyllosphere microbiota obtained from *G. album* leaves of the four independent C plots, but were much more variable for leaf samples derived from the four T plots. Based on the Chao 1 index (representing the total number of phylogenetic groups, **Figure 4A**) it could be shown, that the bacterial richness was more similar in *G. album* leaves grown in C (316–364) compared to T plots (223–646). The richness of phylogenetic groups of the bacterial communities in T plots was higher compared to C plots for two of the T plots (T-1G and T-4G) and lower for the other two T plots (T-2G and T-3G). The composition of the bacterial phyllosphere microbiota of leaves from C plots was less equally distributed (evenness index, **Figure 4B**) because of the high abundance of individual phylogenetic groups as indicated by high dominance values for C samples (**Figure 4C**). The dominance and evenness measurements of the microbial communities of the T plots again were highly variable among each other. Generally, the phyllosphere bacterial communities were much more equally distributed, without single abundant taxa and more diverse (Shannon index; **Figure 4D**) compared to C plots in two of the T plots (T-1 and T-4; **Figures 4B–D**). The opposite was obtained for the phyllosphere microbiota derived from the other two T plots (T-2 and T-3). The differences of the bacterial communities of the leaf samples from the four T plots among each other and in comparison to the C plots were also illustrated by the differences in the dynamics of the rarefaction curves (**Figure 4E**). Rarefaction curves of T samples had always a higher slope than the curves of the C samples indicating the presence of more complex bacterial communities under elevated temperature conditions.

### Phylogenetic Groups with Strong Impact on Differences in the Phyllosphere Microbiota under Control and Warmed Conditions

**FIGURE 5:**
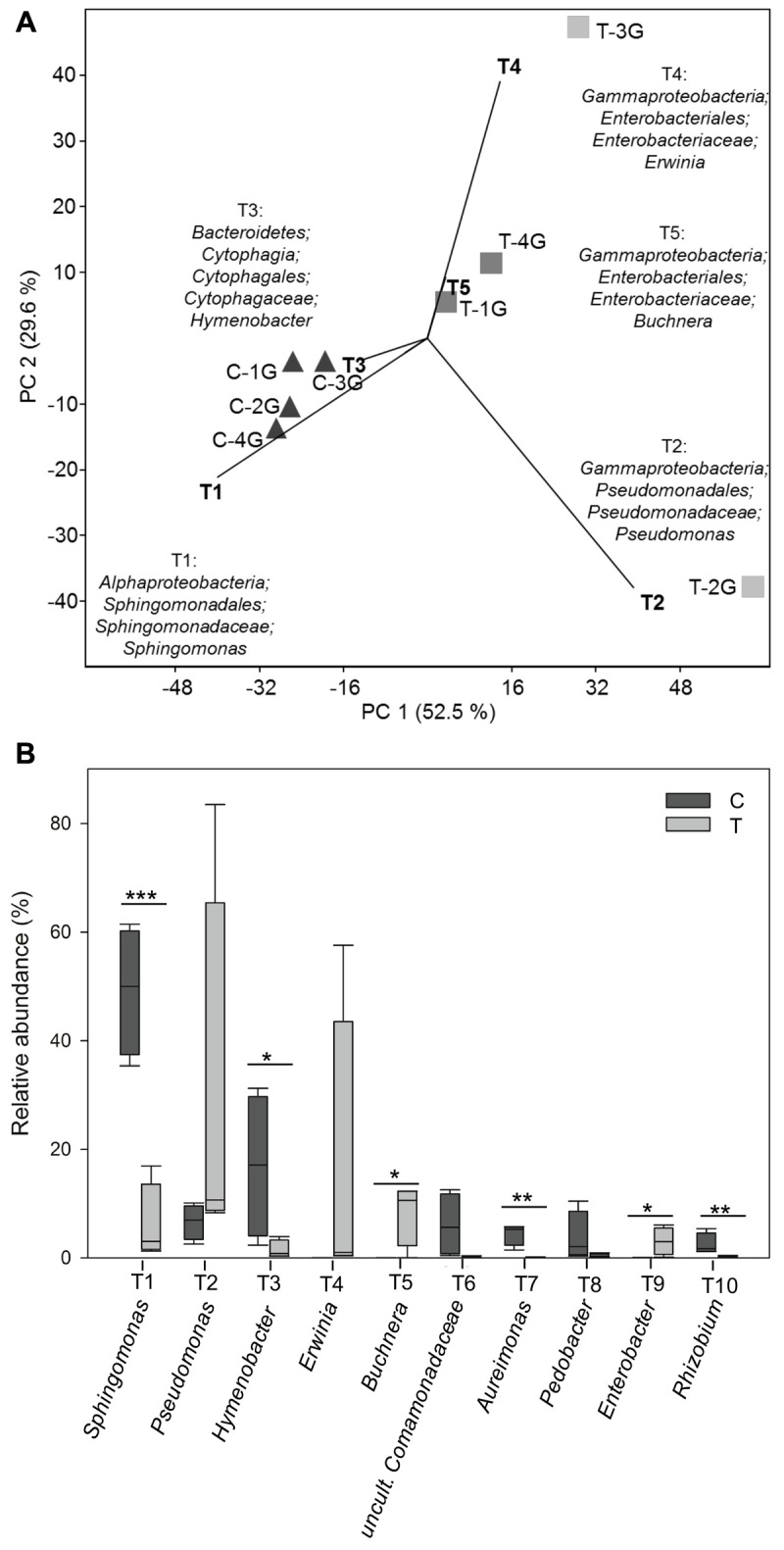
**(A)** Principal component analysis (PCA) of relative abundance pattern of respective bacterial communities from *Galium album* leaves based on the taxa assignment. Eigenvalues for the compared principal components were given in brackets (%) at the respective axes of the graphs. The contribution of different taxonomic groups to the placement of the samples in the PCA plots are indicated as biplots in the graph. Different taxa were numbered with T1-T111 in accordance with their contribution to the differences between leaf microbiota from C and T plots; T1 represents the taxa with the highest contribution. **(B)** Relative abundance of the most abundant 10 taxa present on *Galium album* leaves from C and T plots. Asterisks are representing statistical significance: ^∗^*p* < 0.05; ^∗∗^*p* < 0.01; ^∗∗∗^*p* < 0.001.

Principal component analysis (**Figure 5A**) of bacterial community patterns with a resolution at the level of phylogenetic groups and a respective similarity percentage (SIMPER, Supplementary Table 4) analysis were performed to determine the contribution of individual phylogenetic groups to the differences between bacterial communities obtained from control and warmed plots. In addition, *t*-tests were performed for each phylogenetic group to determine those, which were significantly impacted by warming. A total of 62 phylogenetic groups were found, which showed a significant change along with warming (**Figure 5B** and Supplementary Table 5).

**FIGURE 6:**
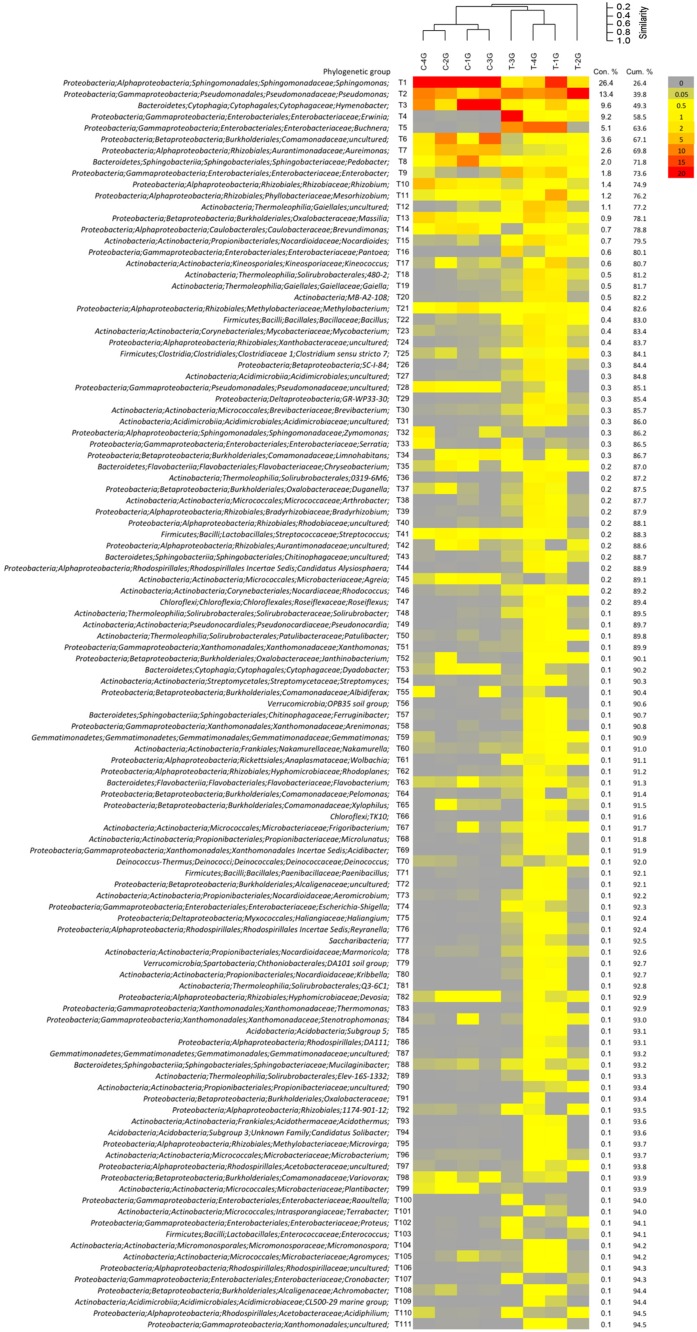
Relative abundance pattern of bacterial taxa present on *Galium album* leaves grown in control (C) and warming (T) plots. Analysis were performed at the level of taxonomic paths (resolved up to the genus level). Community pattern were compared by cluster analysis in PAST3 using UPGMA as clustering algorithm and Bray–Curtis similarity calculation. Taxa were sorted by their contribution to the differentiation between leaf microbiota from C and T plots by the SIMPER analysis using PAST. Data are shown for taxa with ≥0.1% contribution.

Biplots in the PCA analysis (**Figure 5A**) and box plots of the ranking based on the contribution to the differences on the bacterial community patterns (SIMPER) (**Figures 5B**, **6** and Supplementary Table 4) showed, that 10 phylogenetic groups contributed with 75% to the differences between the phyllosphere microbiota of *G. album* leaves derived from C and T plots.

Phylogenetic groups, which had the main contribution to the differences among the microbial communities on leaves of C and T plots were *Proteobacteria* and *Bacteroidete*s. Four of the 10 main contributing phylogenetic groups which occurred with significant higher relative abundance under control conditions were *Sphingomonas* (tax ID T1; *Alphaproteobacteria*), *Hymenobacter* (T3, *Bacteroidetes*), *Aureimonas* (T7), and *Rhizobium* (T10) (both *Alphaproteobacteria*). Two phylogenetic groups also occurred in higher relative abundances under control conditions. They were uncultured *Comamonadaceae* (T6, *Betaproteobacteria*) and *Pedobacter* (T8, *Bacteroidetes*), but differences were not significant. The other four main contributing phylogenetic groups occurred in a higher relative abundance on leaves of T plots were *Gammaproteobacteria*: *Pseudomonas* (T2), *Erwinia* (T4), *Buchnera* (T5), and *Enterobacter* (T2). Differences in relative abundances were only significant for *Buchnera* and *Enterobacter*, because *Pseudomonas* and *Erwinia* occurred only in a high abundance in leaf samples derived from one of the four T plots, T-2G (83.5%) and T-3G (57.6%), respectively, and in lower relative abundance in leaf samples of the other three T plots (9.8 ± 1.2% and 0.8 ± 0.4%, respectively). The high relative abundance of those two phylogenetic groups in T-3G and T-2G was the main reason for the differences in diversity of two T plot samples compared to the other two T plots.

### Effects of Elevated Temperature on Phyla-Specific Bacterial Communities

Diversity measurements and community profiles were considered for the most abundant phyla including *Proteobacteria* (separated into *Alpha-*, *Beta-*, *Delta-*, and *Gammaproteobacteria*), *Bacteroidetes*, *Actinobacteria*, and *Firmicutes* (Supplementary Figures 3–10).

#### Bacteroidetes

The total relative abundance of *Bacteroidetes* was negatively affected by warming (**Figure 2C** and Supplementary Figure 3a). The dominance index was significantly higher in C plots, because of the high relative abundance of two genera, *Hymenobacter* (T3) and *Pedobacter* (T8) under control conditions. The low relative abundance of those taxa in T plots led to a significantly higher diversity of *Bacteroidetes* on *G. album* leaves grown under elevated temperature (Supplementary Figure 3b). *Hymenobacter* and *Pedobacter*, contributed mostly to the differences between the *G. album* leaf microbiota from C and T plots (Supplementary Figure 3c). The boxplots of the ranked distance analysis demonstrated a high variability within the community composition in C and T plots (Supplementary Figure 11).

#### Alphaproteobacteria

The total relative abundance of *Alphaproteobacteria* was also negatively affected by warming (**Figure 2C** and Supplementary Figure 4a). Significantly more single dominant phylogenetic groups, *Sphingomonas* (T1), *Aureimonas* (T7), and *Rhizobium* (T10), were detected in control compared to warmed plots (all *p* < 0.05; Supplementary Figures 4b,c). Due to the lower relative abundance of those genera, the diversity increased significantly under warmed conditions (Supplementary Figure 4b). *Mesorhizobium* (T11) occurred in high abundance on leaves from T plots (especially high in one replicate: T-1G) and contributed also to the differences between *G. album* leaf microbiota from C and T plots (Supplementary Figure 4c). The *Alphaproteobacteria* community compositions showed significant differences with respect to the similarities within C and T plot replicates. The *Alphaproteobacteria* communities were markedly more similar within control plots compared to warmed plots as shown by ranked distance analysis (Supplementary Figure 11).

#### Gammaproteobacteria

In contrast to the *Alphaproteobacteria*, the total relative abundance of *Gammaproteobacteria* was positively affected by warming (**Figure 2C** and Supplementary Figure 5a). In three of four warmed plots, *Gammaproteobacteria* were more equally distributed with less single dominant phylogenetic groups. Differences in the diversity indices were not significant here (Supplementary Figure 5b). Within this subclass the genera *Pseudomonas* (T2), *Erwinia* (T4), *Buchnera* (T5), and *Enterobacter* (T9) contributed most to the differences between the *G. album* leaf microbiota from control and warmed plots and each of them were abundant on leaves from T plots (Supplementary Figure 5c). Ranked distance analysis showed more similarity of the *Gammaproteobacteria* community within C plots and a significant higher variability of the community in T plots (Supplementary Figure 11).

#### Betaproteobacteria

Warming had no significant effect on the total relative abundance of *Betaproteobacteria* communities (**Figure 2C** and Supplementary Figure 6a). Uncultured *Comamonadaceae* (T6) and *Massilia* (T13) were detected in high abundance in control plots. The dominance index was significantly higher under C compared to T (Supplementary Figure 6b). In consequence their absence led to a significantly more diverse community in the leaf phyllosphere of warmed plots (Supplementary Figure 6b). The phylogenetic groups assigned to SC-I-84 (T26) and *Duganella* (T37) were abundant in T plots and had together with T6 the highest contribution to the differences between leaf microbiota from C and T plots (Supplementary Figure 6c). The ranked distance analysis showed a high variability of *Betaproteobacteria* within C and T plots (Supplementary Figure 11).

#### Deltaproteobacteria

The total relative abundance of *Deltaproteobacteria* was not influenced by warming (**Figure 2C** and Supplementary Figure 7a). In three of the four warmed plots, less single dominant phylogenetic groups, but a higher diverse community of *Deltaproteobacteria* was detected (Supplementary Figure 7b). The diversity indices were not significantly different. Within this subclass GR-WP33-30 (T29), *Haliangium* (T75), P3OB-42 (T138), uncultured *Sandaracinaceae* (T185) and group BIrii41 (T113) contributed mainly to the differences between the *G. album* leaf microbiota from C and T plots and were all abundant on leaves from T plots (Supplementary Figure 7c). The variability of the community was smaller within T plots compared to C plots (Supplementary Figure 11).

*Actinobacteria* and *Firmicutes* were the two main phyla, which occurred in significantly higher relative abundance in warmed plots (**Figure 2C**). Single dominant phylogenetic groups dominated *Actinobacteria* and *Firmicutes* communities of leaves from C plots, but not from T plots, which led to a higher diversity of *Actinobacteria* and *Firmicutes* under warmed conditions (Supplementary Figures 8a–j, 9a, and 10a, respectively).

Within the *Actinobacteria*, the genus *Agreia* (T45) was dominant under control conditions (Supplementary Figure 9b). Uncultured members of the *Gaiellales* (T12), *Nocardioides* (T15), *Kineococcus* (T17), uncultured group 480-2 (T18), *Gaiella* (T19), uncultured group MB-A2-108 (T20), *Mycobacterium* (T23) and *Brevibacterium* (T30) were abundant on leaves from T plots (Supplementary Figure 9b). All nine phylogenetic groups contributed to the differences between the leaf microbiota from C and T plots. Ranked distance analysis showed, that the community patterns were more similar among C plots and had a high variability among T plots (Supplementary Figure 11).

Four main phylogenetic groups of *Firmicutes* had the strongest impact on the differences between control and warmed plots (Supplementary Figure 10b). *Streptococcus* spp. (T41) were abundant under C. *Bacillus* (T22), *Clostridium sensu stricto* 7 (T25) and *Paenibacillus* (T71) were detected with a high relative abundance in T plots. Ranked distance analysis showed a high variability of *Firmicutes* within control and warmed plots with a higher variability under elevated temperature (Supplementary Figure 11).

## Discussion

Our study showed that surface warming did not strongly affect the abundance of bacteria in the phyllosphere but had several effects on the diversity and phylogenetic composition of the phyllosphere inhabiting bacterial communities. Bacterial richness and diversity increased with higher temperature. Ren et al. (2015) also reported an increase in bacterial richness and diversity in the rice leaf phyllosphere with the combined effects of elevated CO_2_ and temperature (eCO_2_+eT), but no effects were determined by analyzing each factor separately. Campisano et al. (2017) also showed, that higher growth temperatures (35°C compared to 15°C) led to a higher diversity of endophytic bacterial communities in above ground organs of *Vitis vinifera* plants grown under controlled conditions in greenhouse experiments.

The relative abundance and composition of the dominating bacterial phyla, *Proteobacteria, Bacteroidetes*, and *Actinobacteria*, were affected differently (**Figure 2A**). These three phyla were often described as phyllosphere-associated generalists occurring as most abundant phyla in the phyllosphere of different plant species (Bulgarelli et al., 2013; Bringel and Couée, 2015). The occurrence of these three phyla in the phyllosphere was also determined in several other studies (Kim et al., 2012; Rastogi et al., 2012; Horton et al., 2014; Bai et al., 2015; Dees et al., 2015). To understand the effects of surface warming on the bacterial microbiota in detail, required an in-depth study on the effects of the relative abundance of individual bacterial taxa within the abundant but also less abundant phyla, which were also significantly affected by warming, e.g., the *Firmicutes.* As also indicated by the genome sequence based study of leaf-derived bacterial isolates performed by Bai et al. (2015), different leaf associated members of the *Actinobacteria* for example had similar biological functions while the functions among different members of the *Firmicutes* were much more complex. Shifts of individual taxa can therefore strongly affect the functionality of the phyllosphere microbiota with respect to plant–microbe as well as microbe–microbe interactions. At present, the knowledge of functional traits of phyllosphere bacteria is limited and mainly focused on the culturable fraction of the leaf microbiota.

### Effects of Warming on Common Abundant Phyllosphere Inhabiting Bacteria

Several well-known phyllosphere inhabiting bacterial genera such as *Sphingomonas*, *Hymenobacter*, *Aureimonas, Pedobacter*, and *Rhizobium*, had the main impact on the differences of the community profiles of leaf samples from C and T plots. All were negatively influenced with respect to their relative abundance by warming. The potential functions of those phylogenetic groups and the reason and consequence for changes in their relative abundance will be discussed in more detail:

#### Sphingomonas

Differences in the relative abundance of *Sphingomonas* (tax ID T1, **Figures 5**, **6**) made the highest contribution (26.4%; **Figure 6**) to the differences between the phyllosphere microbiota of *G. album* leaves from C and T plots. *Sphingomonas* occurred in a high relative abundance on leaves grown in C plots (49.2 ± 10.3%) and in a lower relative abundance on leaves grown in T plots (6.0 ± 6.3%; Supplementary Table 4). *Sphingomonas* are well-known as phyllosphere inhabiting bacteria of different plants and for their plant protective potential against pathogens (Delmotte et al., 2009; Innerebner et al., 2011; Vorholt, 2012). Ren et al. (2015) found by a 16S rRNA gene based 454 pyrosequencing approach, that *Sphingomonadaceae*, which includes *Sphingomonas* as type genus, showed a slight reduction under elevated temperature in the rice phyllosphere. Their lower relative abundance on plants grown under elevated temperature may cause a negative effect on plant protection and in consequence enhance the growth of plant pathogens. In a laboratory study of Innerebner et al. (2011) it was shown, that the population size of the plant pathogens *Pseudomonas syringae* pv. tomato DC3000 and *Xanthomonas campestris* pv. *campestris* LMG 568 was reduced on *Arabidopsis* leaves after leaf inoculation with a *Sphingomonas* sp. strain, that was isolated previously from the phyllosphere of grasses.

The lower relative abundance of *Sphingomonas* in the leaf phyllosphere of *G. album* grown in T plots may be explained by changes in the substrate availability on *G. album* plants grown under elevated temperature. Metaproteome studies of the phyllosphere microbiota showed a high abundance of sugar uptake transporters of *Sphingomonas* which indicated, that plant-released carbohydrates are the main carbon source of phyllosphere inhabiting *Sphingomonas* spp. (Delmotte et al., 2009). Jansen-Willems et al. (2016) showed, that the plant biomass increased in T plots, which indicated changes of the plant physiology and morphology. A first insight into metabolome profiles of *G. album* leaves of C and T plots indicated significant differences among leave metabolomes (unpublished data). The impact of changed substrate profiles of the plants in general will be an important aspect for the shift of the phyllosphere microbiota. Bai et al. (2015) showed in a comparative genomic study of phyllosphere and root derived bacterial isolates, that phyllosphere bacteria contain a significantly higher fraction of annotated proteins linked to the carbohydrate and amino acid metabolism, indicating the importance of changes in substrate availability for phyllosphere inhabiting microbiota. If the changed substrate pattern available under elevated temperature did not match the optimal substrate utilization capacity of *Sphingomonas*, those may have been outcompeted by other phylogenetic groups, which could more efficiently use these substrates and occupy that novel ecological niche characterized by specific substrate profiles.

#### Hymenobacter

The second most abundant phylogenetic group, the genus *Hymenobacter* was significantly less abundant in the phyllosphere of *G. album* in warmed plots (tax ID T3, **Figures 5**, **6**). It occurred with a high relative abundance in the *G. album* phyllosphere of C plots (C: 16.9 ± 11.7%) and with only 1.4 ± 1.5% relative abundance in the phyllosphere of *G. album* grown in T plots (Supplementary Table 4). The genus *Hymenobacter* contributed with 9.6% to the differences between the *G. album* leaf microbiota from C and T plots. Several studies showed that members of the genus *Hymenobacter* are common inhabitants of the phyllosphere (Ottesen et al., 2009; Leveau and Tech, 2011), but little is known about their function. Ding and Melchner (2016) described *Hymenobacter* as dominant endospheric bacteria in leaf samples from five plant species (*Ambrosia psilostachya* DC., *Asclepias viridis* Walt., *Panicum virgatum* L., *Sorghastrum nutans* (L.) Nash, and *Ruellia humilis* Nutt.).

Related to the microbial “hub” concept discussed by Agler et al. (2016), genera which occurred in a high relative abundance in the phyllosphere under control conditions, as *Sphingomonas* and *Hymenobacter*, may represent “hub” taxa within the phyllosphere microbiota of *G. album* plants. Their low relative abundance in the phyllosphere of *G. album* leaves grown under elevated temperature may be one reason for the high variability or high instability of the T microbiota, which was especially indicated by increased abundance of individual members of the *Gammaproteobacteria* including potential pathogenic genera, *Pseudomonas* or *Erwinia*. Copeland et al. (2015) described members of the genus *Sphingomonas* as leaf adapted taxa and *Pseudomonas* and *Erwinia* as taxa with moderate prevalence and variable abundance on leaves of different crop plants. Based on the microbial “hub” concept, the loss of central hub taxa may disturb the stability of the phyllosphere microbiota, if those strongly interacting taxa are functionally not replaced. Other taxa, which include potentially pathogenic bacteria may be able to grow in ecological niches which were previously occupied by the “hub” taxa without replacing their functionality, i.e., the high abundance of different *Gammaproteobacteria* in warmed plots. The localization of *Hymenobacter* in or at the surface of leaves of *G. album* is so far not known, but may be endospheric as indicated by Ding and Melchner (2016). Thereby *Hymenobacter* may be a “hub” taxon, which occupies another ecological niche than the mainly surface attached *Sphingomonas* (Delmotte et al., 2009; Vorholt, 2012).

#### Aureimonas

Amplicon sequences assigned to the genus *Aureimonas* were also detected in significantly lower abundance on *G. album* leaves grown under elevated temperature with a relative abundance between 4.4 (±1.7)% in C and 0.08 (±0.03)% in T plots (**Figures 5B**, **6** and Supplementary Table 4). The contribution of this genus to the differences between the phyllosphere microbiota from C and T plots was 2.6% (**Figure 6**). Little is known and reported about the genus *Aureimonas* and its occurrence in the phyllosphere. The genus *Aureimonas* belongs to the *Aurantimonadaceae*, which are very closely related to the genus *Rhizobium*, which contains many plant growth-promoting *bacteria*. Among the currently described ten species with validated names, four were isolated from the phyllosphere. *A. galii* and *A. pseudogalii* (Aydogan et al., 2016) were both isolated from *G. album* leaves, the type stain of *A. galii* even from leaves of the current experiment from plants grown under C. Strains of the same species were not cultured from leaves of plants grown under T, which supported the finding of the reduced abundance of this genus in warmed plots (data not shown). *A. phyllosphaerae* and *A. jatrophae* (Madhaiyan et al., 2013) were isolated from surface-sterilized leaf tissues of *Jatropha curcas* L. cultivars, which indicated an endophytic life style.

#### Pedobacter

The second phylogenetic group of *Bacteroidetes*, which was among the ten phylogenetic groups that showed main contribution to the differences among the bacterial community composition under warmed conditions and occurred with a slightly lower but not significant relative abundance in warmed plots, was the genus *Pedobacter* (**Figures 5B**, **6**). *Pedobacter* spp. was detected also in other phyllosphere studies (Nissinen et al., 2012; Vorholt, 2012; Humphrey et al., 2014; Dees et al., 2015). *Pedobacter* infection was negatively correlated with herbivory and may affect plant–microbe–herbivore interactions in a similar manner as specific *Pseudomonas* spp. (*P. fluorescence*) (Humphrey et al., 2014).

#### Rhizobium

Sequences assigned to the genus *Rhizobium* were also detected in significant lower relative abundance on *G. album* leaves grown in T (0.2 ± 0.2%) compared to C plots (2.5 ± 1.7%) (**Figure 5B** and Supplementary Table 4). They contributed with 1.4% to the differences between the leaf microbiota from C and T plots (**Figure 6**). Members of the genus *Rhizobium* were often reported as phyllosphere inhabiting bacteria (Wellner et al., 2011; Knief et al., 2012; Horton et al., 2014) with beneficial effects on the plant phyllosphere (Nandi et al., 1982). *Rhizobium* sp. are well known for their plant growth promotion and induction of systemic resistances, which positively affects plant fitness (Glaeser et al., 2016).

### Warming Effects on Potential Plant and/or Human Pathogens

#### Pseudomonas

The genus *Pseudomonas* (tax ID T2, **Figures 5**, **6**) had the second strongest contribution (13.4%) to the differences among the microbial community patterns of leaves after warming, due to an increased relative abundance of *Pseudomonas* on leaves derived from T plots (28.2 ± 31.9%) compared to C plots (6.6 ± 2.7%, Supplementary Table 4). The high abundance of sequences assigned to the genus were however, only detected on the leaf phyllosphere from one (T-2G: 83.5%) of four T plots with significantly higher abundance compared to C plots. The high abundance of *Pseudomonas* in one warmed plot sample was the main reason for the high dominance index value obtained for this T sample (**Figure 4C**), and had also a strong contribution to the high variability among the total bacterial community patterns of T plots (**Figure 3B**). Differences of the relative abundance of *Pseudomonas* were therefore not significant between the leaf microbiota from C and T plots. The genus *Pseudomonas* was often reported as a dominating phyllosphere inhabitant (Lopez-Velasco et al., 2011; Rastogi et al., 2012; Bodenhausen et al., 2013). Within the genus, some species are known as plant or human pathogens (Vorholt, 2012) while other species are well known for their plant protective function (Cronin et al., 1997; Mendes et al., 2011). Mendes et al. (2011) showed, that the presence of plant pathogenic fungi (*Rhizoctonia solani*) enhanced the abundance of the antagonistically acting *Pseudomonadaceae* haplotypes, and illustrated herewith the huge functional diversity even among strains of *Pseudomonas*. An increase in the abundance of distinct *Pseudomonas* spp. (especially *P. syringae*) was also positively correlated, whereas *P. fluorescens* infection was negatively correlated with herbivory (Humphrey et al., 2014). Positive or negative impacts of *Pseudomonas* in the phyllosphere were however, always strain dependent and could strongly vary among different *Pseudomonas* spp. isolated from plants (Mendes et al., 2011; Humphrey et al., 2014). The function of *Pseudomonas* present in the phyllosphere of *G. album* under control and warmed conditions needs to be further analyzed through an investigation of *Pseudomonas* spp. isolates obtained from the respective samples in parallel to the cultivation independent study (data not shown).

#### Enterobacteriaceae

The phyllosphere microbiota of leaves derived from T plots contained a significantly higher abundance of the genus *Enterobacter* (**Figure 5B**). *Enterobacter* spp. contributed with 1.8% (**Figure 6**) to the differences between C (0.03 ± 0.02%) and T (3.0 ± 2.2%) derived phyllosphere bacterial communities, which was also shown with the respective biplot (tax ID T9, *Enterobacter*, **Figure 5A**). The genus *Erwinia* (biplot T4; **Figure 5A**) also had a strong contribution (9.6%) to the differences between leaf microbiota from C and T plots. *Erwinia* spp. occurred with a high relative abundance on *G. album* leaves obtained from one T plot (T-3G: 57.6%, Supplementary Table 4) of four, which led again to the strong variability of the bacterial community composition among leaf samples from elevated temperature plots (**Figure 3B**) and explained also the high dominance index value for the community composition on leaves from this plot (**Figure 4C**). Several species within the genus *Erwinia* are known to be plant pathogens (Starr and Chatterjee, 1972). Other low abundant members of the *Enterobacteriaceae* (*Pantoea, Proteus, Escherichia-Shigella, Serratia*) occurred in slightly higher abundances on *G. album* leaves from T plots, and other genera like *Buttiauxella, Citrobacter, Cronobacter, Klebsiella, Kluyvera, Pectobacterium, Providencia, Raoultella, Tatumella*, and uncultured *Enterobacteriaceae* were detected only on leaves grown in T plots (Supplementary Table 4) although the results were not statistically significant because of the variability within the bacterial community on leaves obtained from T plots. *Enterobacteriaceae* in general constituted 2.6% of all detected phylogenetic groups. In summary, the data suggests that an increase of the surface temperature by +2°C had a long-term effect on the microbial community of the *G. album* leaf phyllosphere with an increase of the relative abundance of *Enterobacteriaceae*. Rastogi et al. (2012) detected more *Enterobacteriaceae* and culturable coliforms in summer compared to winter samples on field grown lettuce leaves, and Ren et al. (2015) also detected slightly higher *Enterobacteriaceae* in the upper rice leaf phyllosphere with elevated temperature.

#### Other Potential Pathogens

A significantly higher relative abundance of potential plant and/or human pathogens, as classified by the “Technische Regel für Biologische Arbeitsstoffe” (TRBA 466) for Germany as risk group 2 microbes, like members of *Curtobacterium, Clostridium, Peptoclostridium, Aeromonas, Rhodococcus, Acinetobacter, Burkholderia, Bacillus, Staphylococcus, Cellulomonas, Corynebacterium* and a slightly but not significantly higher relative abundance of the potential plant pathogen *Xanthomonas* were detected on leaves of *G. album* from T plots (Supplementary Tables 4, 5). In former studies it is already reported, that plants represent reservoirs for potential plant or human pathogens like *Pseudomonas, Staphylococcus, Enterococcus, Burkholderia*, and *Enterobacteriaceae* (Berg et al., 2014; Schikora and Schikora, 2014). Sydnor and Perl (2011) described genera like *Acinetobacter*, *Enterobacter*, *Klebsiella* (*K. pneumonia, K. oxytoca*), *Proteus*, *Pseudomonas*, and *Serratia* as antibiotic-resistant microorganisms, which play an important role in hospital-acquired infections. *Acinetobacter baumannii* is regarded as an emerging pathogen and one of most important multidrug-resistant bacteria in hospitals worldwide (Peleg et al., 2008; Antunes et al., 2014). These findings indicate a potential risk for an increase in potential plant and/or human pathogens on *G. album* leaves with elevated temperature due to global warming.

### Indication of Warming Effects on Arthropods and/or Nematodes

#### Buchnera

*Galium album* leaf phyllosphere from elevated temperature plots harbored a significantly higher abundance of sequences assigned to *Buchnera* (C: 0.002 ± 0.002%; T: 8.3 ± 5.0%; **Figure 5** and Supplementary Table 4) with a contribution of 5.1% to the differences between the leaf microbiota from C and T plots (**Figure 6**). The strong impact of the high relative abundance of *Buchnera* on the microbial community differences among both treatments was also indicated by the respective biplot (**Figure 5A**; *Buchnera*, T5). *Buchnera* spp. are well known endosymbionts of aphids (Moran et al., 2008). The high relative abundance of *Buchnera* in the warmed plots therefore indicated an enhanced occurrence of aphids on leaves of *G. album* in T plots, as shown in the Supplementary Figure 12 for a *G. album* plant from the same field side. Either aphids or *Buchnera* only were coextracted with plant material. Aphids constitute a globally distributed agricultural pest as sap-sucking herbivores, using large amounts of phloem as a nutritional source (Gray and Fraenkel, 1954). Our findings were in congruence with the results obtained by Barton and Ives (2014). They detected an increased aphid population growth rate on sweet corn grown under elevated temperature (elevated daily temperature of 4.87°C ± 0.14°C). Dong et al. (2013) also described, that experimental warming led to an increase in pest aphid abundance on wheat fields.

#### Wolbachia

The increase of the relative abundance of insects or at least the enhanced relative abundance of insect symbionts was demonstrated for the genus *Wolbachia*. However, *Wolbachia* spp. occurred generally only in a low abundance. A significantly (*p* ≤ 0.01, Student’s *t*-test) higher relative abundance of *Wolbachia* was detected in the samples analyzed from T (0.2 ± 0.1%) compared to C (0.001 ± 0.001%) plots (Supplementary Tables 4, 5). *Wolbachia* spp. are intracellular bacteria found in ∼40% arthropods and filarial nematodes (Ferri et al., 2011; Zug and Hammerstein, 2012). Most of the filarial nematodes can cause dangerous filariasis (river blindness and elephantiasis) in humans, and arthropods can lead to dengue fever, yellow fever, malaria and Chikungunya virus (as reviewed by Slatko et al., 2014). Both of them, arthropods and nematodes, can be found in the phyllosphere (Erb et al., 2008; as reviewed by Lindow and Brandl, 2003; Müller et al., 2016).

As reviewed by Robinet and Roques (2010) there is an impact of global warming on several insects, which respond with earlier flight periods, enhanced winter survival and acceleration of development rates. In contrast, a long-term monitoring study of insects feeding on mountain birch (*Betula pubescens* ssp. *czerepanovii*) demonstrated, that climate change influenced insect herbivory in heavily stressed compared to pristine forests differently, and that herbivorous insects showed diverse responses to climate variations (Kozlov et al., 2017). Kozlov et al. (2017) highlighted the problems of predicting effects of global change on forest damaging insects, which requires long-term observations.

The higher relative abundance of *Buchnera* and *Wolbachia* on leaves of *G. album* were an indicator of increased numbers of arthropods and filarial nematodes with elevated temperature, which can represent a vector for potential human diseases (Tobias, 2016).

### Warming Effects on Plant Beneficial Methylotrophs Often Dominating the Phyllosphere

#### Methylobacterium

Highly abundant and plant beneficial bacterial members of the phyllosphere microbiota are members of the genus *Methylobacterium* (Delmotte et al., 2009; Wellner et al., 2011; Knief et al., 2012; Vorholt, 2012). *Methylobacterium* spp. normally represent the most abundant methylotrophic bacteria. Sequences assigned to the genus *Methylobacterium* were only detected in low abundance (<0.1%, Supplementary Table 4) in the phyllosphere of *G. album* leaves. A non-significantly lower relative abundance of *Methylobacterium* sequences was observed with elevated temperature. One important reason for the low detection rate of *Methylobacterium* sequences may be due to the difficulties in the DNA extraction from this genus. Reisberg et al. (2012) mentioned the same problem by analyzing DNA extracts of the *Arabidopsis* phyllosphere. However, pure culture test experiments showed that DNA from *Methylobacterium* spp. could be efficiently extracted with the DNA extraction kit applied here for the leaf samples. Whether or not the relative abundance of *Methylobacterium* sp. in the *G. album* phyllosphere is common, needs to be investigated further.

## Conclusion

This is a first systematic study showing that global warming can have significant effects on the phyllosphere inhabiting bacterial communities caused by surface warming predicted within the next decades as consequence of global climate change. *G. album* was used as model plant and showed changes in the abundances of well-known members of plant phyllosphere bacteria in general and may therefore be representative for many land plants. Important plant protective and beneficial bacteria like members of the *Sphingomonas* and *Hymenobacter* were negatively affected with respect to their relative abundance. The loss of key or “hub” taxa may lead to a more imbalanced phyllosphere microbiota composition, which may then become more susceptible for a stronger colonization with plant and human pathogens. Significantly higher abundances of, e.g., *Enterobacteriaceae*, *Pseudomonas*, or *Acinetobacter* indicate that moderate surface warming may cause a increase of potentially pathogenic bacteria on leaves. The colonization of plants with those bacteria is also problematic due to the fact that antibiotic resistances, linked to respective taxa, may be distributed into the environment.

The increase of insect symbionts in the sequence datasets obtained from warming plots supports the findings of other studies, that surface warming increases the abundance of plant associated insects. Differences in carbon source patterns presented in leaves, but also changes in the leaf associated microbiota may contribute to the increase of insect abundance. However, the increased insect abundance acting as transmission vectors may fasten plant damage in agroecosystems and, thus, may also increase the risk for the spread of plant and human pathogens (Thomas, 2017). This can play a role along with global climate change and needs to be considered in further studies.

This study provided clear evidence that surface warming by +2°C will have a strong impact on the bacterial phyllosphere microbiota. Warming leads to an increase in ecosystem photosynthesis (see review by Wu et al., 2011) which effects the availability of carbon sources in the phyllosphere. Changes in the availability of carbon substrates will be in general one of the key factors contributing to changes in the composition of the phyllosphere microbiota. Competition for substrates is often one of the main drivers in shaping microbial community compositions. The loss of abundant hub taxa may play an important role in the changes of the composition of phyllosphere microbiota. In addition, changes of environmental parameters may have also contributed to the microbiota shift. Jansen-Willems et al. (2016) determined a net loss in soil carbon in the T plots caused by an increased soil respiration with elevated temperature. As Luo (2007) discussed, changes in the soil and the increased aboveground biomass can also lead to plant stress caused by the higher water and nutrient demands of the plant, which may have impacted the leaf microbiota. However, this is not confirmed by other studies so far. Further studies are required to explain the effects in more detail and to estimate positive and negative effect of surface warming on the phyllosphere microbiota and in consequence on plants and also humans.

## Author Contributions

SG and EA designed the project, analyzed and interpreted the data, and wrote the manuscript; GM, SG, and EA did the field sampling; EA performed the laboratory experiments; GM and CM were responsible for the long-term field experiment; PK got funding for the project; PK, GM, and CM made final approval of the manuscript.

## Conflict of Interest Statement

The authors declare that the research was conducted in the absence of any commercial or financial relationships that could be construed as a potential conflict of interest.

## Footnotes

1 http://bbmap.sourceforge.net/

2 https://www.arb-silva.de/ngs/

3 http://www.bioinformatics.org/cd-hit

4 http://www.arb-silva.de

5 http://blast.ncbi.nlm.nih.gov/Blast.cgi
