# Supplementary material for: Long-Term Warming Shifts the Composition of Bacterial Communities in the Phyllosphere of Galium album in a Permanent Grassland Field-Experiment
Source: Front Microbiol. 2018 Feb 13;9:144. doi: 10.3389/fmicb.2018.00144 (PMC5816784; doi:10.3389/fmicb.2018.00144)
Supplement: Supplementary file 1 [file Table_1.DOCX]

**Supplementary Table 1** Concentration of total cells detached from the leaf surface of *Galium album* leaves with PBS buffer. Ten digital images of SG-I stained cells were generated with a Leica DFC 3000G (Leica, Germany) camera system and cells were counted. The mean and standard deviation of the 10 picture counts were calculated. The mean of the four Control (C) and warming (T) plots were calculated with using error propagation.

| Plots | Mean (± standard propagation) |
| --- | --- |
| C-G | 4.0 (± 1.4) x 10^5^ |
| T-G | 3.9 (± 1.3) x 10^5^ |

| Plots | Mean (± standard deviation) |
| --- | --- |
| C-1G | 2.7 (± 0.4) x 10^5^ |
| C-2G | 4.1 (± 0.7) x 10^5^ |
| C-3G | 4.9 (± 0.9) x 10^5^ |
| C-4G | 4.3 (± 0.7) x 10^5^ |
| T-1G | 4.3 (± 0.7) x 10^5^ |
| T-2G | 2.8 (± 0.5) x 10^5^ |
| T-3G | 3.9 (± 0.7) x 10^5^ |
| T-4G | 4.6 (± 0.7) x 10^5^ |
