# Supplementary material for: Long-Term Warming Shifts the Composition of Bacterial Communities in the Phyllosphere of Galium album in a Permanent Grassland Field-Experiment
Source: Front Microbiol. 2018 Feb 13;9:144. doi: 10.3389/fmicb.2018.00144 (PMC5816784; doi:10.3389/fmicb.2018.00144)
Supplement: Supplementary file 2 [file Table_2.DOCX]

**Supplementary Table 2** Relative abundance of chloroplast, mitochondria, *Archaea* and non-related sequence in the final data set of the Illumina 16S rRNA gene amplicon sequencing, the minimal (min) and maximal (max) sequence amount per sample and the final percentage (%) of total sequences.

| Groups | Sequences | min sequences per sample | max sequences per sample | % of total |
| --- | --- | --- | --- | --- |
| *Bacteria* (final data set) | 433,922 | 7,627 | 88,073 | 73.5 |
| *Archaea* | 304 | 0 | 219 | 0.1 |
| Chloroplast | 113,103 | 169 | 45,893 | 19.1 |
| Mitochondria | 42,530 | 186 | 16,345 | 7.2 |
| Non-related | 787 | 21 | 276 | 0.1 |
