# Supplementary material for: Long-Term Warming Shifts the Composition of Bacterial Communities in the Phyllosphere of Galium album in a Permanent Grassland Field-Experiment
Source: Front Microbiol. 2018 Feb 13;9:144. doi: 10.3389/fmicb.2018.00144 (PMC5816784; doi:10.3389/fmicb.2018.00144)
Supplement: Supplementary file 6 [file Presentation_1.pdf]

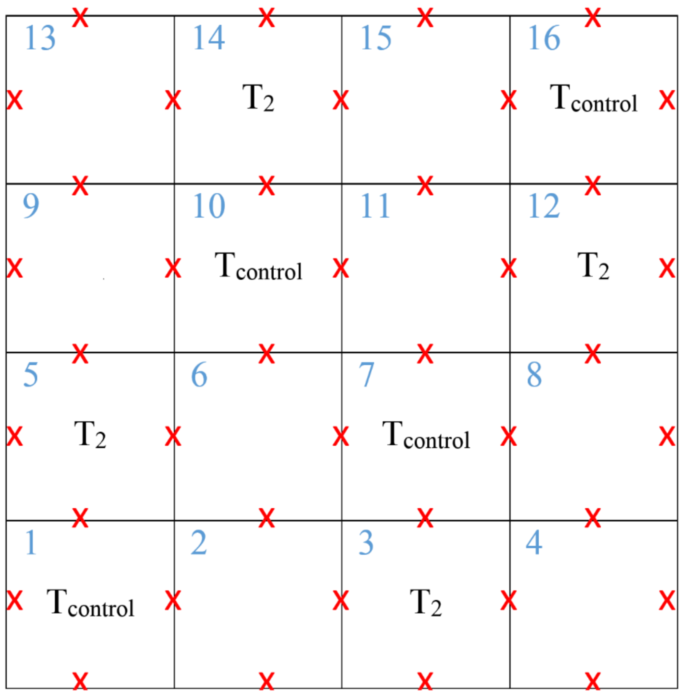

Supplementary Figure 1 Experimental setup. Four treatments were assigned to 16 plots according to a Latin square. The numbers are the plot numbers. T was heated to 2°C above ambient temperature and C was not heated. The crosses show the soil sampling locations in 2007.

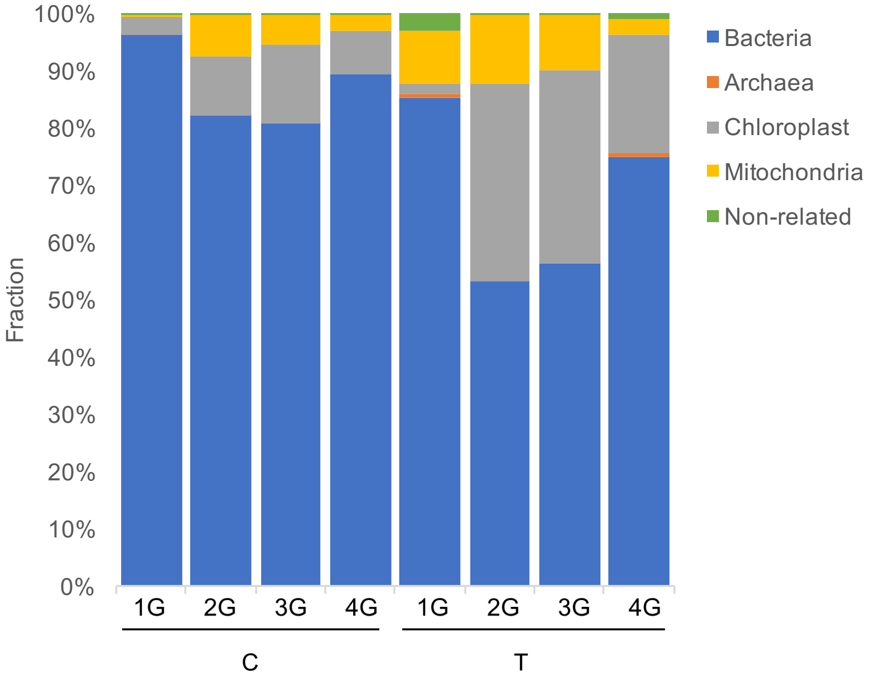

Supplementary Figure 2 Fraction (%) of chloroplast, mitochondria, *Archaea* and non-related reads in the 16S rRNA gene amplicon sequences obtained from the individual *G. album* leaf samples of C and T plots.

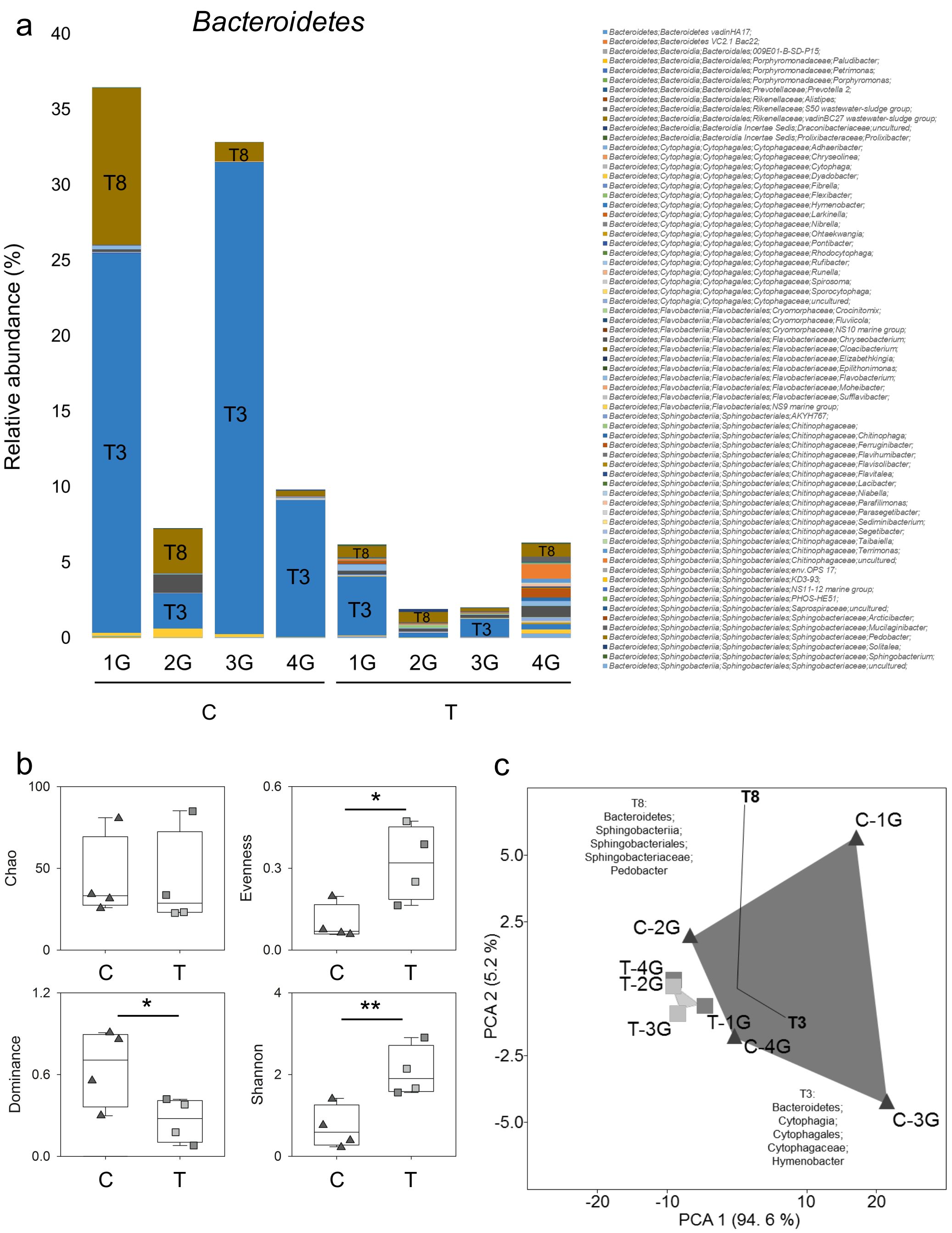

Supplementary Figure 3 a, Phylogenetic composition of phyllosphere inhabiting *Bacteroidetes* of *Galium album* leaves from plots with ambient temperature (C 1-4) and +2 °C surface temperature elevated plots (T 1-4) resolved at the level of *Bacteria* taxa. b, Box-plots of alpha diversity indices Chao 1, evenness, dominance and shannon index of detected *Bacteroidetes* on *Galium album* leaves obtained from C and T plots. c, Principal component (PCA) analysis of relative abundance patterns of *Bacteroidetes* communities from *G. album* leaves based on the taxa assignment. Eigenvalues for the compared principal components were given in brackets (%) at the respective axes of the graphs. The contribution of different taxonomic groups to the placement of the samples in the PCA plots are indicated as biplots in the graph. Different taxa were numbered with T1-T111 in accordance with their contribution to the differences between leaf microbiota from C and T plots, T1 represents the taxa with the highest contribution. Box-plots and student's t-test were performed in Sigmaplot (Applied Maths). Asterisks are representing statistical significance: *=$p < 0.05$; **=$p < 0.01$; ***=$p < 0.001$.

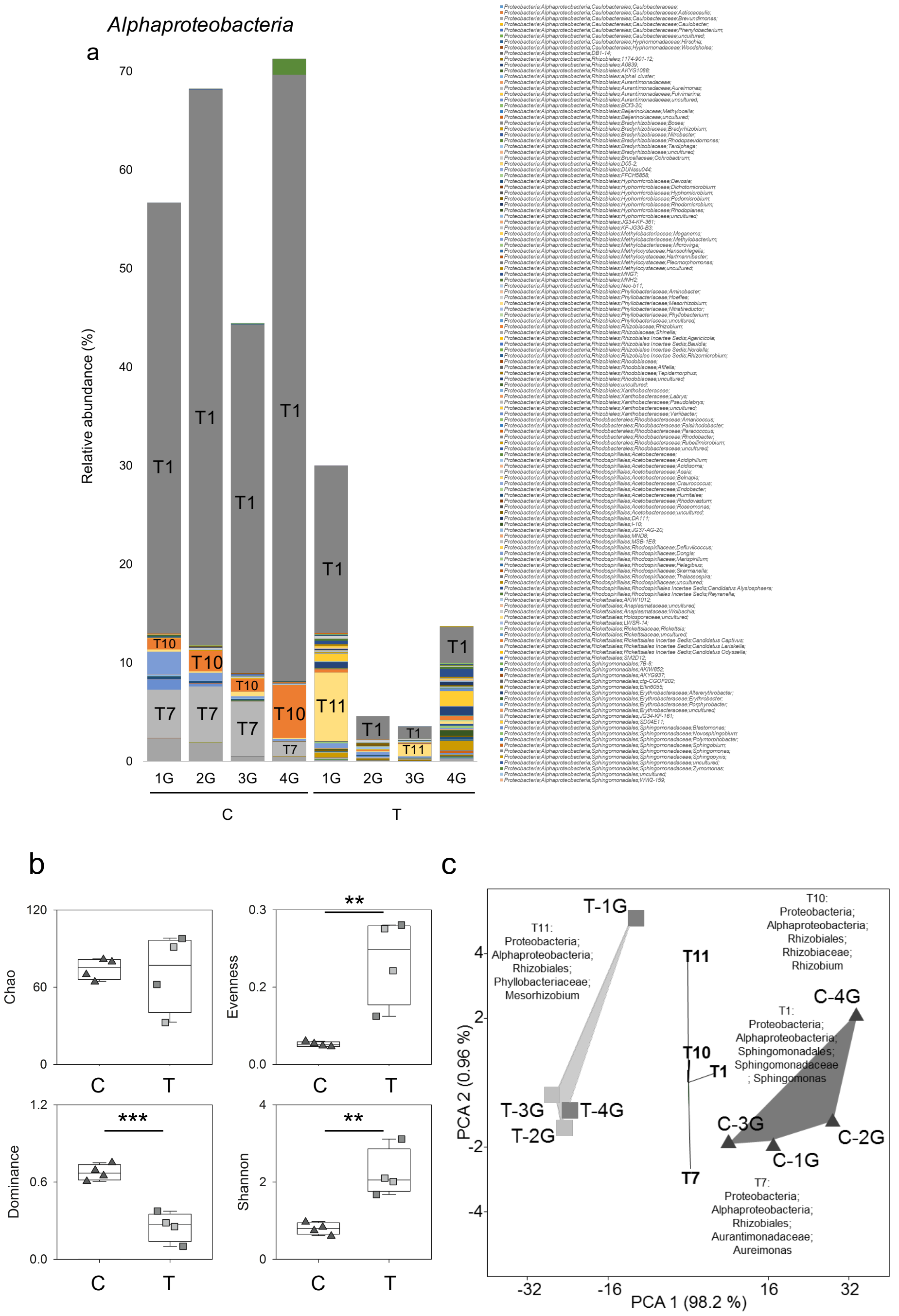

Supplementary Figure 4 a, Phylogenetic composition of phyllosphere inhabiting *Alphaproteobacteria* of *Galium album* leaves from plots with ambient temperature (C 1-4) and +2 °C surface temperature elevated plots (T 1-4) resolved at the level of *Bacteria* taxa. b, Box-plots of alpha diversity indices Chao 1, evenness, dominance and shannon index of detected *Alphaproteobacteria* on *Galium album* leaves obtained from C and T plots. c, Principal component (PCA) analysis of relative abundance patterns of *Alphaproteobacteria* communities from *G. album* leaves based on the taxa assignment. Eigenvalues for the compared principal components were given in brackets (%) at the respective axes of the graphs. The contribution of different taxonomic groups to the placement of the samples in the PCA plots are indicated as biplots in the graph. Different taxa were numbered with T1-T111 in accordance with their contribution to the differences between leaf microbiota from C and T plots, T1 represents the taxa with the highest contribution. Box-plots and student's t-test were performed in Sigmaplot (Applied Maths). Asterisks are representing statistical significance: *=$p < 0.05$; **=$p < 0.01$; ***=$p < 0.001$.

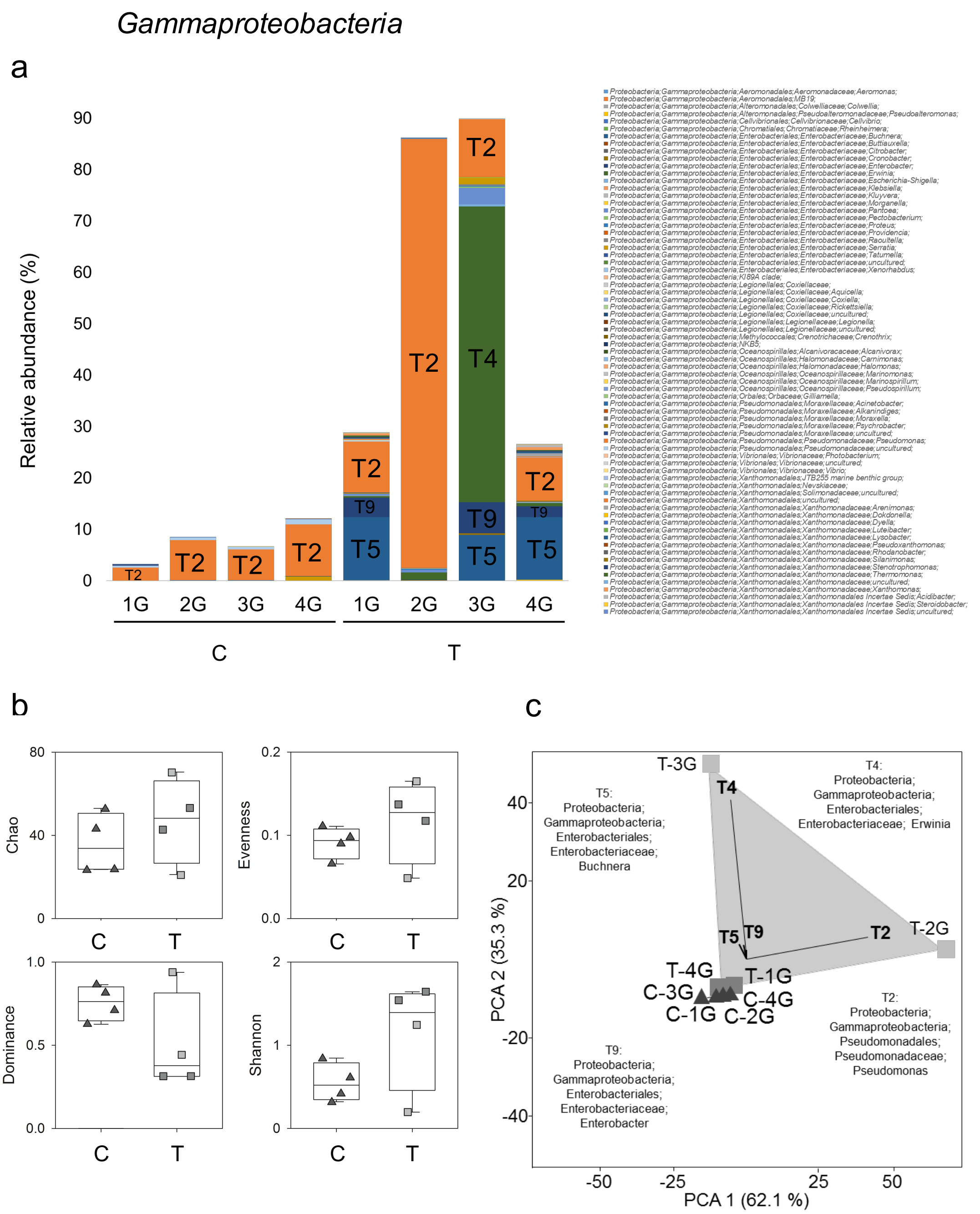

Supplementary Figure 5 a, Phylogenetic composition of phyllosphere inhabiting *Gammaproteobacteria* of *Galium album* leaves from plots with ambient temperature (C 1-4) and +2 °C surface temperature elevated plots (T 1-4) resolved at the level of *Bacteria* taxa. b, Box-plots of alpha diversity indices Chao 1, evenness, dominance and shannon index of detected *Gammaproteobacteria* on *Galium album* leaves obtained from C and T plots. c, Principal component (PCA) analysis of relative abundance patterns of *Gammaproteobacteria* communities from *G. album* leaves based on the taxa assignment. Eigenvalues for the compared principal components were given in brackets (%) at the respective axes of the graphs. The contribution of different taxonomic groups to the placement of the samples in the PCA plots are indicated as biplots in the graph. Different taxa were numbered with T1-T111 in accordance with their contribution to the differences between leaf microbiota from C and T plots, T1 represents the taxa with the highest contribution. Box-plots and student's t-test were performed in Sigmaplot (Applied Maths).

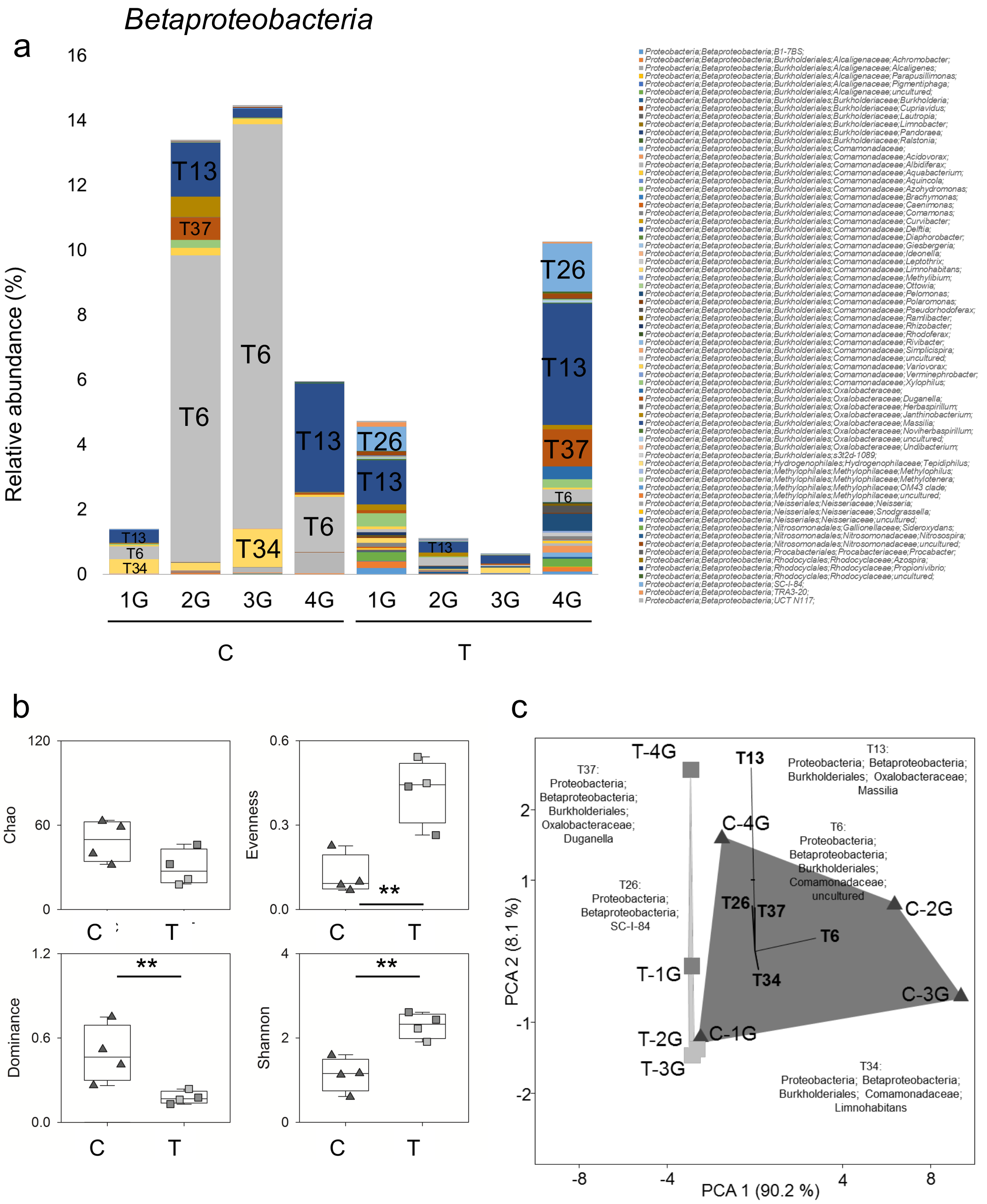

Supplementary Figure 6 a, Phylogenetic composition of phyllosphere inhabiting *Betaproteobacteria* of *Galium album* leaves from plots with ambient temperature (C 1-4) and +2 °C surface temperature elevated plots (T 1-4) resolved at the level of *Bacteria* taxa. b, Box-plots of alpha diversity indices Chao 1, evenness, dominance and shannon index of detected *Betaproteobacteria* on *Galium album* leaves obtained from C and T plots. c, Principal component (PCA) analysis of relative abundance patterns of *Betaproteobacteria* communities from *G. album* leaves based on the taxa assignment. Eigenvalues for the compared principal components were given in brackets (%) at the respective axes of the graphs. The contribution of different taxonomic groups to the placement of the samples in the PCA plots are indicated as biplots in the graph. Different taxa were numbered with T1-T111 in accordance with their contribution to the differences between leaf microbiota from C and T plots, T1 represents the taxa with the highest contribution. Box-plots and student's t-test were performed in Sigmaplot (Applied Maths). Asterisks are representing statistical significance: *=p < 0.05; **=p < 0.01; ***=p < 0.001.

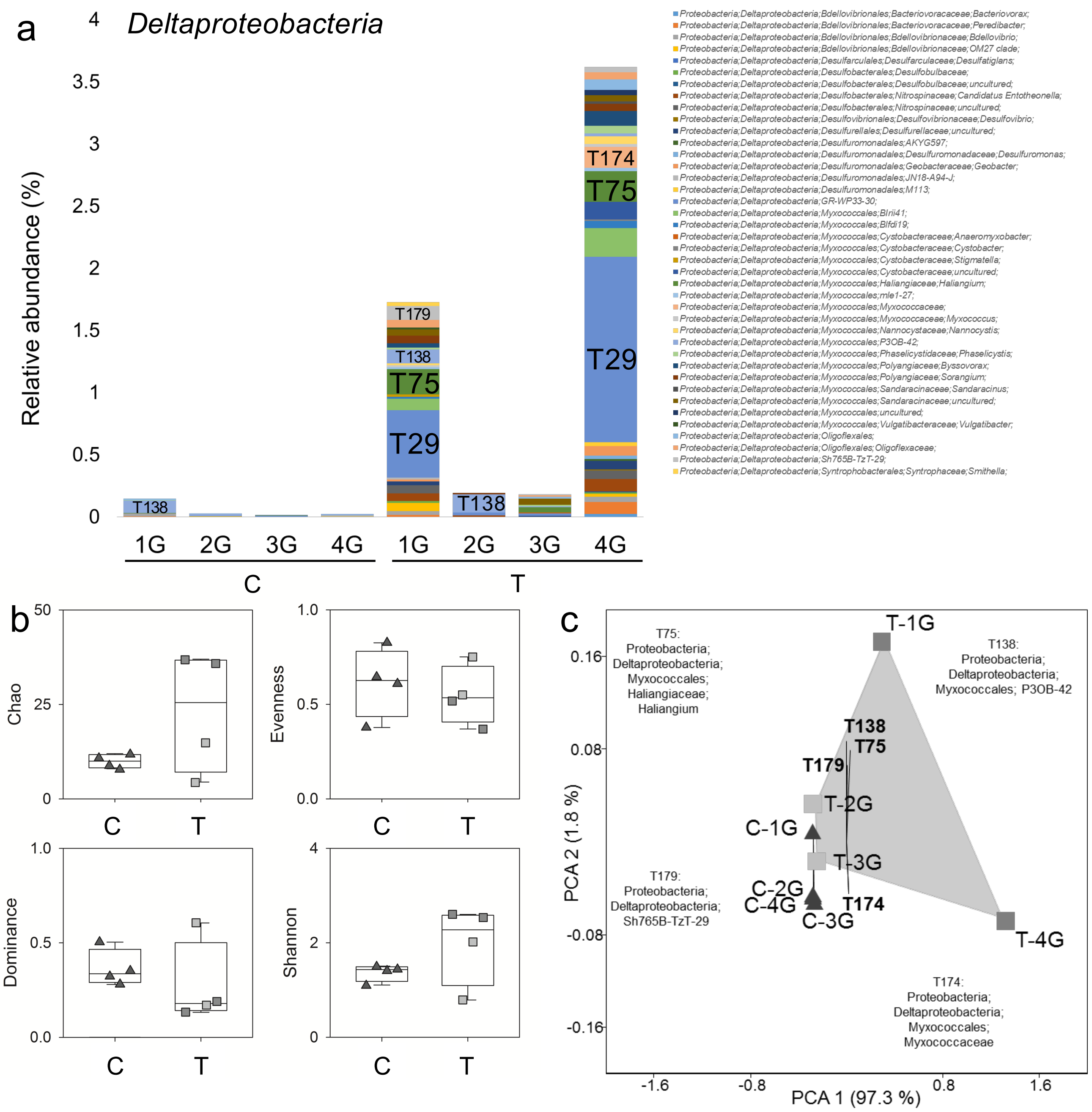

Supplementary Figure 7 a, Phylogenetic composition of phyllosphere inhabiting *Deltaproteobacteria* of *Galium album* leaves from plots with ambient temperature (C 1-4) and +2 °C surface temperature elevated plots (T 1-4) resolved at the level of *Bacteria* taxa. b, Box-plots of alpha diversity indices Chao 1, evenness, dominance and shannon index of detected *Deltaproteobacteria* on *Galium album* leaves obtained from C and T plots. c, Principal component (PCA) analysis of relative abundance patterns of *Deltaproteobacteria* communities from *G. album* leaves based on the taxa assignment. Eigenvalues for the compared principal components were given in brackets (%) at the respective axes of the graphs. The contribution of different taxonomic groups to the placement of the samples in the PCA plots are indicated as biplots in the graph. Different taxa were numbered with T1-T111 in accordance with their contribution to the differences between leaf microbiota from C and T plots, T1 represents the taxa with the highest contribution. Box-plots and student's t-test were performed in Sigmaplot (Applied Maths).

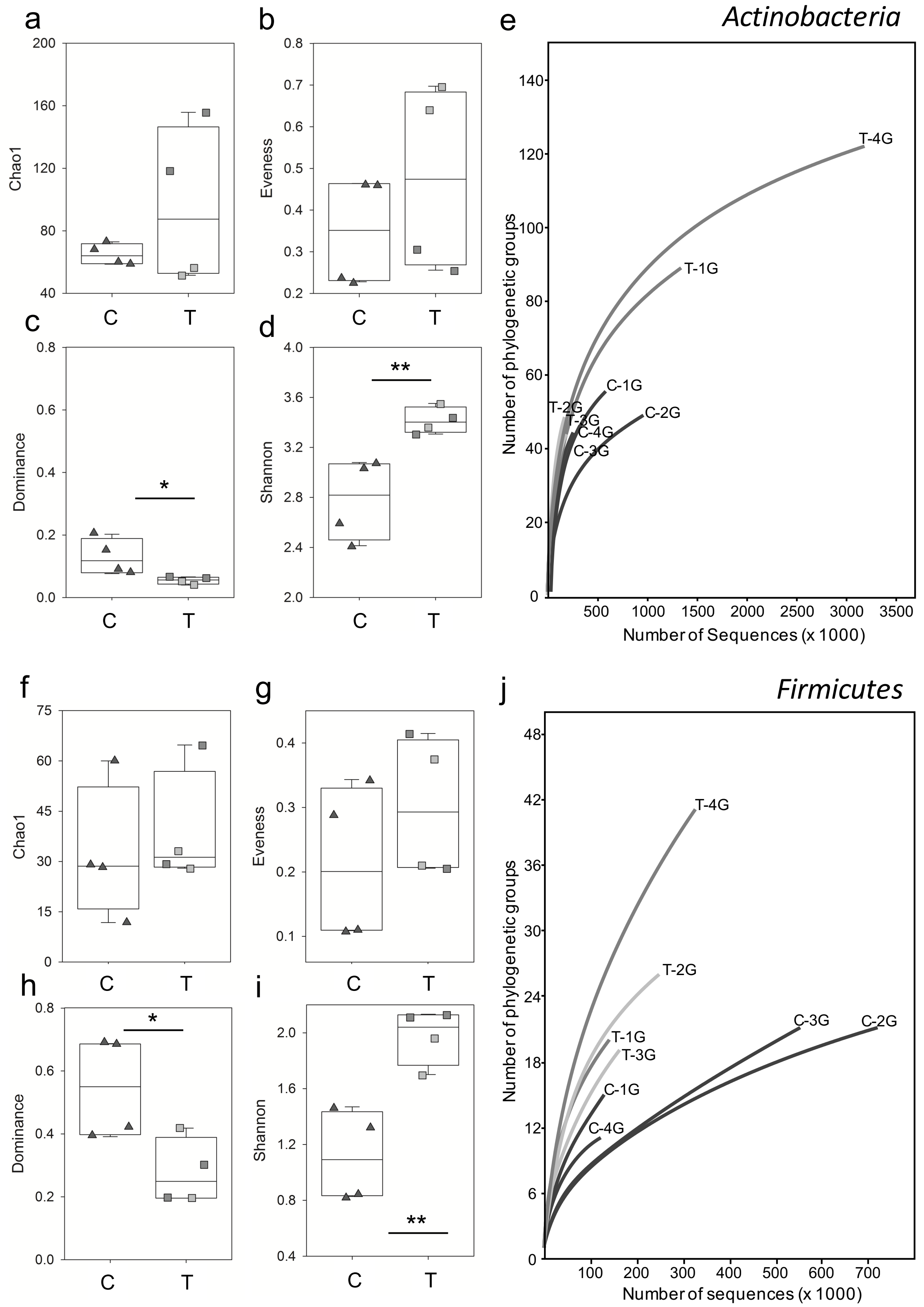

Supplementary Figure 8 Box-plots of alpha diversity indices Chao 1, evenness, dominance and shannon index (a-d) and rarefaction curves (e) of detected *Actinobacteria* and (f-j) *Firmicutes* on *Galium album* leaves obtained from C and T plots. C= ambient temperature (C), T= elevated temperature (T). Squares = four C plots, triangles = four T plots. Box-plots and student's t-test were performed in Sigmaplot (Applied Maths). Asterisks are representing statistical significance: *=p < 0.05; **=p < 0.01; ***=p < 0.001.

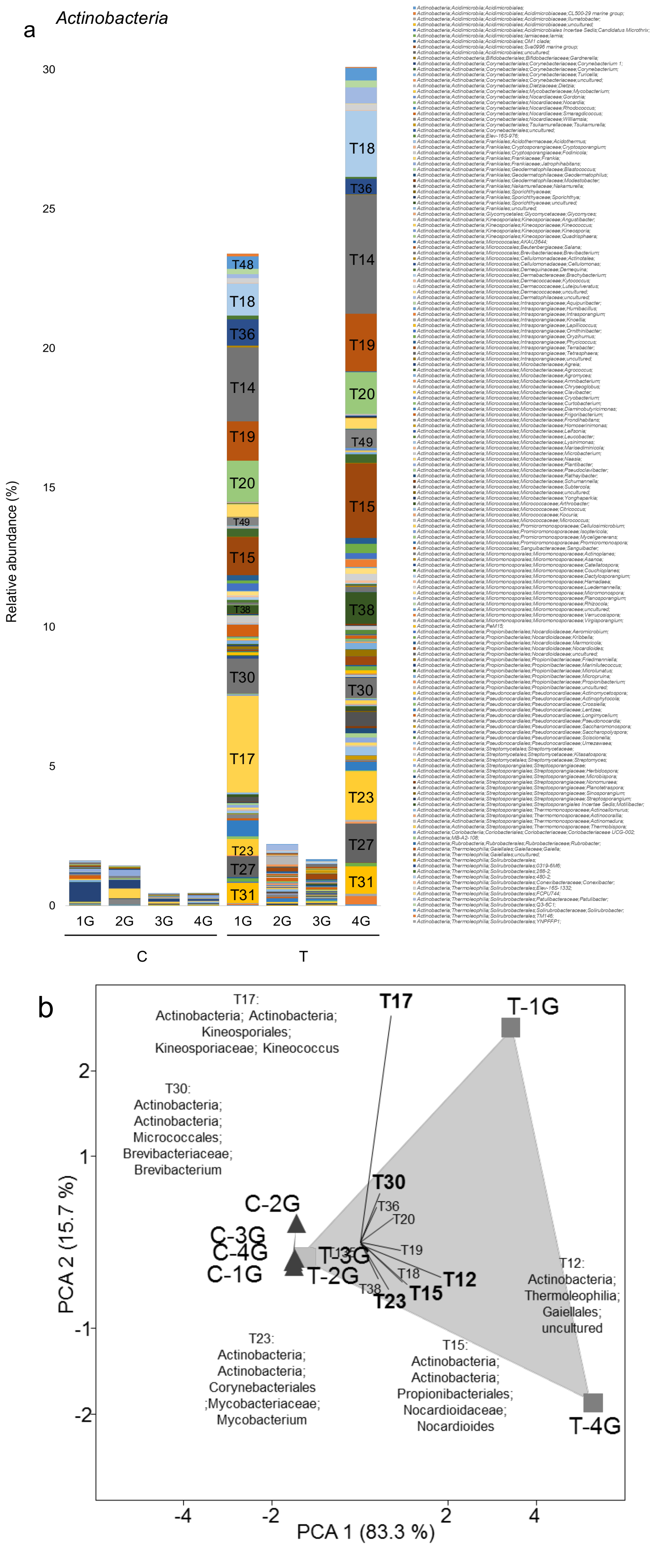
a
Actinobacteria
Relative abundance (%)
0
5
10
15
20
25
30
T48
T18
T36
T14
T19
T20
T49
T15
T38
T30
T17
T23
T27
T31
1G
2G
3G
4G
C
T
b
T17:
Actinobacteria; Actinobacteria;
Kineosporiales;
Kineosporiaceae; Kineococcus
T30:
Actinobacteria;
Actinobacteria;
Micrococcales;
Brevibacteriaceae;
Brevibacterium
T23:
Actinobacteria;
Actinobacteria;
Corynebacteriales
;Mycobacteriaceae;
Mycobacterium
T15:
Actinobacteria;
Actinobacteria;
Propionibacteriales;
Nocardioidaceae;
Nocardioides
T12:
Actinobacteria;
Thermoleophilia;
Gaiellales;
uncultured
T-1G
T-4G
T-3G
T-2G
C-2G
C-3G
C-4G
C-1G
T17
T30
T36
T20
T19
T18
T12
T15
T23
T38
PCA 2 (15.7 %)
PCA 1 (83.3 %)
2
1
-1
-2
-4
-2
2
4

Supplementary Figure 9 a, Phylogenetic composition of phyllosphere inhabiting *Actinobacteria* of *Galium album* leaves from plots with ambient temperature (C 1-4) and +2 °C surface temperature elevated plots (T 1-4) resolved at the level of *Bacteria* taxa. b, Principal component (PCA) analysis of relative abundance patterns of *Actinobacteria* communities from *G. album* leaves based on the taxa assignment. Eigenvalues for the compared principal components were given in brackets (%) at the respective axes of the graphs. The contribution of different taxonomic groups to the placement of the samples in the PCA plots are indicated as biplots in the graph. Different taxa were numbered with T1-T111 in accordance with their contribution to the differences between leaf microbiota from C and T plots, T1 represents the taxa with the highest contribution. Box-plots and student's t-test were performed in Sigmaplot (Applied Maths).

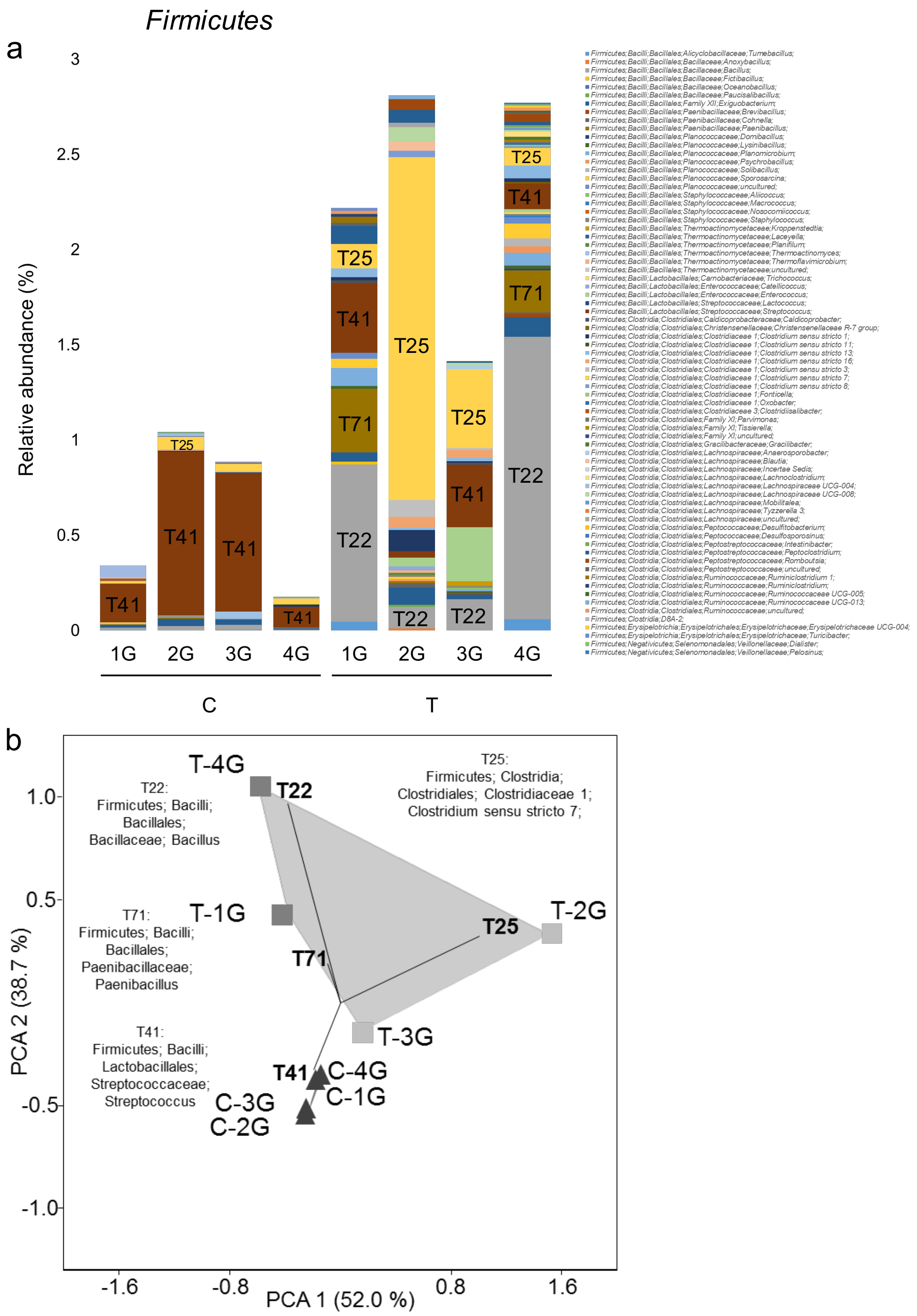

Supplementary Figure 10 Phylogenetic composition of phyllosphere inhabiting *Firmicutes* of *Galium album* leaves from plots with ambient temperature (C 1-4) and +2 °C surface temperature elevated plots (T 1-4) resolved at the level of *Bacteria* taxa. b, Principal component (PCA) analysis of relative abundance pattern of *Firmicutes* communities from *G. album* leaves based on the taxa assignment. Eigenvalues for the compared principal components were given in brackets (%) at the respective axes of the graphs. The contribution of different taxonomic groups to the placement of the samples in the PCA plots are indicated as biplots in the graph. Different taxa were numbered with T1-T111 in accordance with their contribution to the differences between leaf microbiota from C and T plots, T1 represents the taxa with the highest contribution. Box-plots and student’s t-test were performed in Sigmaplot (Applied Maths).

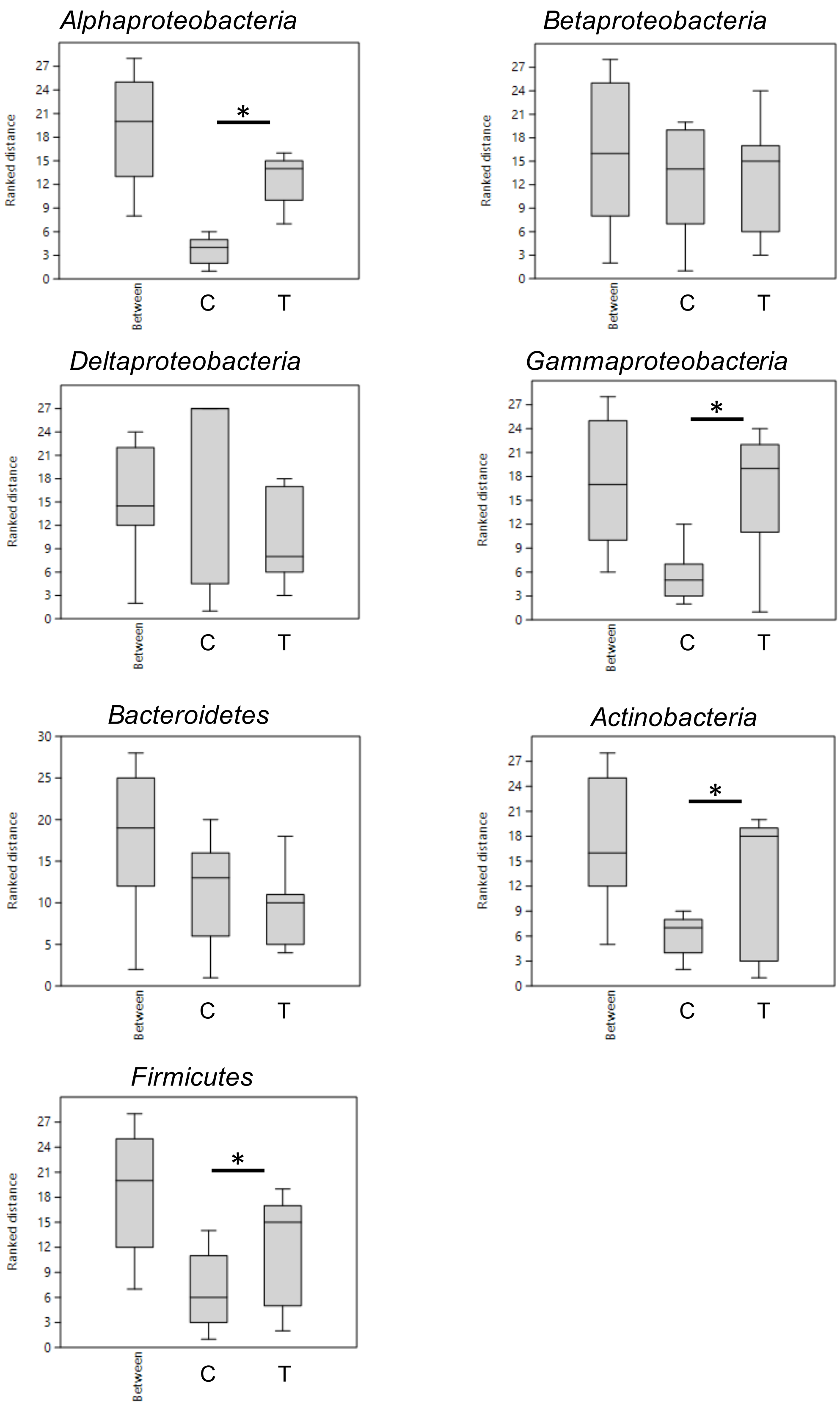

Supplementary Figure 11 Ranked distance analysis of *Alpha-. Beta-, Delta- and Gammaproteobacteria, Bacteroidetes, Actinobacteria* and *Firmicutes* calculated with Bray Curtis similarity matrix and One-way ANOSIM in PAST3. Relative abundance pattern of each phyla from *G. album* leaves based on the taxa assignment. Asterisks are representing statistical significance: *=$p < 0.05$.

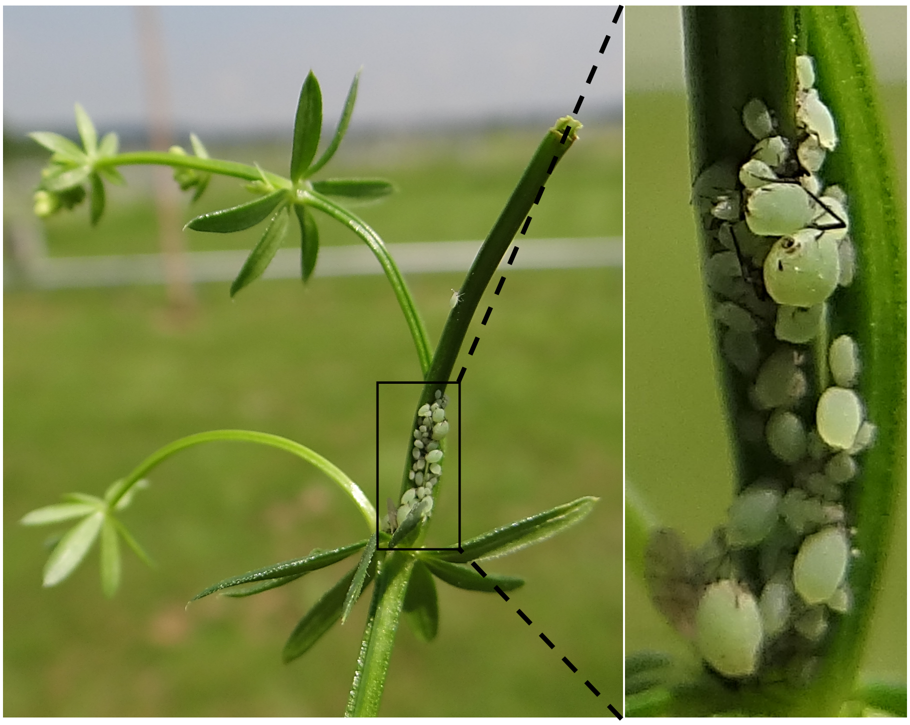

Supplementary Figure 12 *Galium album* plant colonized by aphids and other insects. Picture was taken from the permanent grassland of the Environmental Monitoring and Climate Impact Research Station Linden in Germany.
